# Probiotics for the treatment of ulcerative colitis: a review of experimental research from 2018 to 2022

**DOI:** 10.3389/fmicb.2023.1211271

**Published:** 2023-07-06

**Authors:** Cuilan Huang, Wujuan Hao, Xuyang Wang, Renmin Zhou, Qiong Lin

**Affiliations:** ^1^Wuxi People’s Hospital Affiliated to Nanjing Medical University, Wuxi Children’s Hospital, Wuxi, China; ^2^Department of Digestive, Affiliated Children’s Hospital of Jiangnan University, Wuxi, China

**Keywords:** ulcerative colitis, *Bifidobacterium*, *Lactobacillus*, probiotic, treatment, children

## Abstract

Ulcerative colitis (UC) has become a worldwide public health problem, and the prevalence of the disease among children has been increasing. The pathogenesis of UC has not been elucidated, but dysbiosis of the gut microbiota is considered the main cause of chronic intestinal inflammation. This review focuses on the therapeutic effects of probiotics on UC and the potential mechanisms involved. In animal studies, probiotics have been shown to alleviate symptoms of UC, including weight loss, diarrhea, blood in the stool, and a shortened colon length, while also restoring intestinal microecological homeostasis, improving gut barrier function, modulating the intestinal immune response, and attenuating intestinal inflammation, thereby providing theoretical support for the development of probiotic-based microbial products as an adjunctive therapy for UC. However, the efficacy of probiotics is influenced by factors such as the bacterial strain, dose, and form. Hence, the mechanisms of action need to be investigated further. Relevant clinical trials are currently lacking, so the extension of animal experimental findings to clinical application requires a longer period of consideration for validation.

## 1. Introduction

UC is a type of inflammatory bowel disease (IBD) that manifests as non-specific chronic inflammation of the colonic mucosa with alternating cycles of remission and exacerbation. The annual incidence of UC has risen in recent years, notably among children (Kuenzig et al., 2022). The prevalence of childhood UC varies worldwide, with European countries having the highest prevalence (15.0/100,000), and the prevalence in North America is 10.6/100,000 (Sýkora et al., 2018). However, data regarding the disease from developing and underdeveloped countries is scarce. Approximately 25% of patients with UC develop symptoms before the age of 18, and the prevalence in children over the age of 10 is clearly increasing. One report showed that the age 10–17 subgroup of children made the highest contribution to the increased prevalence (Ye et al., 2020). This points to an increased burden of UC on the health care system as well as patients and caregivers in the future. Abdominal pain, diarrhea, bloody stools, weight loss, and a decrease in bone density and muscle strength are characteristics of UC. All of these symptoms can impede the growth and development of children (Agrawal et al., 2021). Moreover, repeated hospitalizations and disruptions in learning and life can cause psychological changes such as irritability and even depression, which are major obstacles to healthy development (Reed et al., 2021). Eventually, approximately 15% of patients with UC will require surgery within 20 years of diagnosis (Eisenstein, 2018). Additionally, the younger the age at diagnosis, the worse the prognosis and the greater the likelihood of colonic resection and colon cancer, which significantly increase the health risks of children with UC (Agrawal et al., 2021). Therefore, interventions for UC in children are warranted.

**Figure 1:**
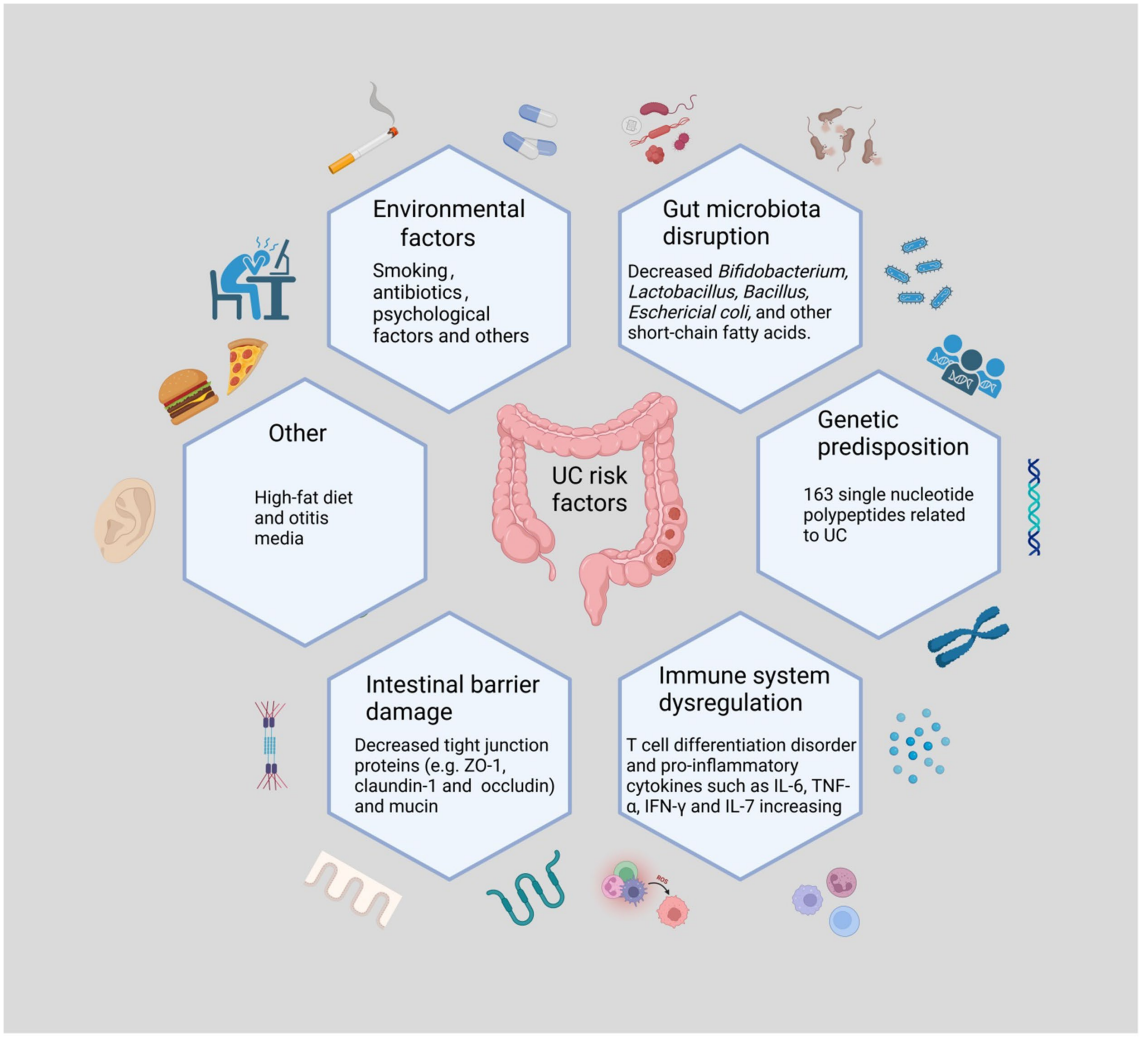
The Main Causes of Ulcerative Colitis. UC may occur as a result of the interaction of the imbalance of gut microbiota, immune disorders, genetic susceptibility, and other factors with external stimuli. The loss of beneficial intestinal flora and the increase of harmful flora disrupt the intestinal environment. The decrease of intestinal epithelial intercellular tight junction proteins as well as mucins also causes damage to the intestinal barrier. Psychological factors and poor lifestyle habits are also risk factors for the development of UC. Various harmful factors trigger intestinal immune disorders and release large amounts of pro-inflammatory factors, causing intestinal inflammation.

The pathogenesis of UC is not entirely known, although it is assumed to be the result of a combination of genetic predisposition, environmental factors, microbial infections, immune system dysregulation, and gut microbiota disruption. This suggests that UC is an immune-related inflammatory disease caused by disturbances in the intestinal environment (Figure 1). Analyzes of the frequency of UC in twins and family members have revealed that being a first-degree relative of an individual with UC is a better predictor of the development of UC than any other environmental factor. Genome-wide association studies revealed 163 single nucleotide polypeptides related to UC and nucleotide binding oligomerization genes (Agrawal et al., 2022; Liu et al., 2023). Environmental factors include smoking, frequent consumption of fast food, antibiotic misuse and abuse, early exposure to antibiotics, and otitis media (Agrawal et al., 2021). Recently, an increasing number of studies have shown dysbiosis of the gut microbiota as the central mechanism in the development of UC (Mizoguchi et al., 2020; Metwaly et al., 2022). A study reporting that pre-existing signs of the imbalance of gut microbiota can be noticed prior to the active phase supports this theory (Kumar and Kumar, 2022). *Clostridium difficile* causes a decrease in beneficial bacteria, a loss of immune function, and increased intestinal permeability. In addition, some studies have shown that transplanting fecal microbiota restores gut ecology, providing a new treatment strategy for UC (Costello et al., 2019; Kelly and Ananthakrishnan, 2019). Lack of protection from beneficial bacteria along with chronic exposure to harmful bacteria causes homeostatic dysbiosis and immunological disturbance, sustaining intestinal inflammation (Oka and Sartor, 2020). Increased numbers of pathogenic microorganisms damage the intestinal epithelial mucus layer, and pathogens that destroy the mucus layer impair the intestinal barrier. After barrier rupture, antigens penetrate the intestinal epithelium, activating downstream mechanistic target of rapamycin (mTOR) via the toll-like receptor (TLR)4 and phosphatidylinositol 3 kinase/protein kinase B (PI3K/AKT) signaling pathways and leading to the production of inflammatory factors, including tumor necrosis factor (TNF)-α, interleukin (IL)-6, and IL-1β (Zhou et al., 2018). In response to antigenic (mostly bacterial) activation, T cells develop into T helper (Th)2, Th9, and Th17 cells, which produce IL-13, IL-9, and IL-17, respectively (Neurath, 2019). Additionally, innate lymphoid cells in the intestine are activated, which then produce TNF-α, interferon (INF)-γ, IL-4, IL-5, IL-17, and IL-22 (Schulz-Kuhnt et al., 2021). Increased levels of inflammatory factors promote neutrophil migration and cell permeability, aggravating the already defective barrier function (Martini et al., 2017), which may also be linked to increased nuclear factor kappa-B (NF-κB) transcription (Peng et al., 2021). Disturbances in the gut microbiota in UC are mostly manifested by a loss of diversity and dominance of *Firmicutes* and *Bacteroidetes*, as well as an increase in the number of *Desulfovibrio* subspecies (Vich Vila et al., 2018; Kumar and Kumar, 2022). This aberrant colony structure reduces levels of short-chain fatty acids (SCFAs), an essential metabolite of bacteria, leading to defective B cell maturation and differentiation as well as lower levels of regulatory cells (Treg), further weakening the mucosal defense (Lavelle and Sokol, 2020). Moreover, bacteria take advantage of the dysbiosis, further perpetuating the vicious cycle. Previous research has revealed complex relationships between inflammatory substances and immune cells, and scientists may uncover new mechanisms of action that are currently unknown.

UC is prone to recurrence after remission. Since there is no cure for UC, treatments are aimed at alleviating symptoms, maintaining remission and improving quality of life. Mainly, treatments can be divided into pharmaceutical and surgical based on the European Crohn’s and Colitis Organization’s treatment recommendations. Biologics, 5-aminosalicylic acid (5-ASA), immunosuppressive drugs, anti-integrin and anti-interleukin antibodies, and topical or systemic steroid hormones are the mainstays of pharmacological therapy (Turner et al., 2018a,b; Raine et al., 2022). Although 5-ASA induces and sustains remission of UC, oral 5-ASA can cause side effects such as stomach discomfort, diarrhea, and nasopharyngitis (Cochrane Gut Group et al., 2020). Additionally, UC is a chronic inflammatory disease that affects patients with impaired gut barrier function. The long-term administration of corticosteroids carries a high risk of complications, such as cataract, glaucoma, and adrenal insufficiency (Valdes and Sobrin, 2020). Biologics are frequently administered to patients who are refractory to previous pharmacological therapies or who are unable to endure severe side effects (Segal et al., 2021). Immunosuppressive drugs and biologics are also advised for individuals with extraintestinal symptoms (Feuerstein et al., 2020; Baumgart and Le Berre, 2021). An increasing number of patients are opting for the early use of immunosuppressive or biological drugs, or both, to increase the chances of remission (Chapman et al., 2020). When administered intermittently, acute infusion reactions occur in 5–10% of cases, and long-term use of immunosuppressive agents can cause side effects such as pancreatitis, leukopenia, nausea, and allergic reactions, as well as increase the risk of infection, heart failure, and cancer (Lemaitre et al., 2017; Singh et al., 2021). Among the biologics, the Food and Drug Administration (FDA) has approved infliximab and adalimumab for pediatric patients with UC, while ustekinumab can only be used in adult patients. Meanwhile, vedolizumab and golimumab are still being studied for use in children (Hyams et al., 2022). Acute, severe, or refractory UC all necessitate surgical intervention such as colorectal resection, fistulae, or flap advancement (Spinelli et al., 2022). Surgical removal of the water-absorbing colorectum can result in severe diarrhea and cause postoperative complications such as intestinal adhesions and obstruction, anastomotic fistula, and abdominal infection. Meanwhile, early surgery in children can affect their growth and development and reduce their quality of life. Although there are several medications available for UC, many patients are ineffective or develop secondary failure during treatment. Due to the above reasons, safe and effective treatments for UC remain limited. Recently, treatment with probiotics has been proposed to regulate the gut microbiome in UC. The gut microbiota plays an essential role in the maintenance of host homeostasis and immunomodulation. Disruptions of inter-species function in gut microbiota contribute to the leading cause of inflammatory bowel disease (Zhang et al., 2022), and UC was demonstrated to be strongly associated with microbial dysbiosis (Kobayashi et al., 2020). The mechanisms of action of probiotics for UC have been extensively researched recently. Hence, probiotics present a potential therapy for pediatric patients with UC due to their safety, low cost, and ease of administration.

Given the pathophysiological importance of the gut microbiome in UC, the hypothesis that altering the gut microbiota may be a viable way to treat UC is gaining attention (Paramsothy et al., 2017). Based on a cross-sectional study, among patients with a high disease activity index (DAI), frequent defecation, severe abdominal pain, and a history of related surgery, the frequency of probiotics being prescribed increased, indicating that probiotics are now being recognized in the treatment of UC (Kim and Cheon, 2022). Although the current evidence base supporting the use of probiotics in patients with UC is thin, probiotics are often widely used as adjunctive therapy and are often recommended by physicians, and they are generally considered to be safe (Abraham and Quigley, 2017). A 2020 Cochrane Review that included 14 studies indicated that probiotics can induce clinical remission during the active period and prevent recurrence in UC patients (Cochrane IBD Group et al., 2020). Additionally, a meta-analysis indicated that VSL#3 resulted in the most significant improvement in UC, followed by *Lactobacillus* and *E. coli* (Shen et al., 2014). However, probiotics have not yet demonstrated a meaningful benefit in maintaining clinical remission in patients with UC (Iheozor-Ejiofor et al., 2020). In children, an analysis of three trials revealed that the combination of *Lactobacillus* with VSL#3 (composed of four different strains of *Lactobacillus* spp., three strains of *Bifidobacterium* spp., and a sole strain of *Streptococcus spp.*) probiotics had significant effects in children with UC (Ganji-Arjenaki and Rafieian-Kopaei, 2018). Another previous study showed that the addition of VSL#3 to regular treatment markedly reduced relapse rates compared with placebo (21.4% vs. 73.3%) when delivered within a year of induction of remission in 29 children with UC (Miele et al., 2009). Unfortunately, there is a lack of clinical studies on the therapeutic effects of probiotics in UC, with most studies being at the animal experimental stage, and there are no compliance randomized controlled trials of probiotics in children with UC; therefore, recommendations for the use of probiotics are not available (Su et al., 2020). Probiotics like *Bifidobacterium* and *Lactobacillus* are recommended as adjuvant therapy in China for adults with mild-to-moderate UC to maintain remission. The European Society for Parenteral Enteral Nutrition also affirms the induction of remission by specific strains of bacteria in patients with mild to moderate UC (Bischoff et al., 2022). Probiotics are highly tolerated and cause few side effects; therefore, it is worth promoting them as a potentially novel treatment alternative for patients with UC (Cochrane IBD Group et al., 2020; Selvamani et al., 2022). Meanwhile, caution should be exercised when applying the results of animal experiments to the treatment of children with UC.

## 2. Therapeutic effects of probiotics on UC

**Figure 2:**
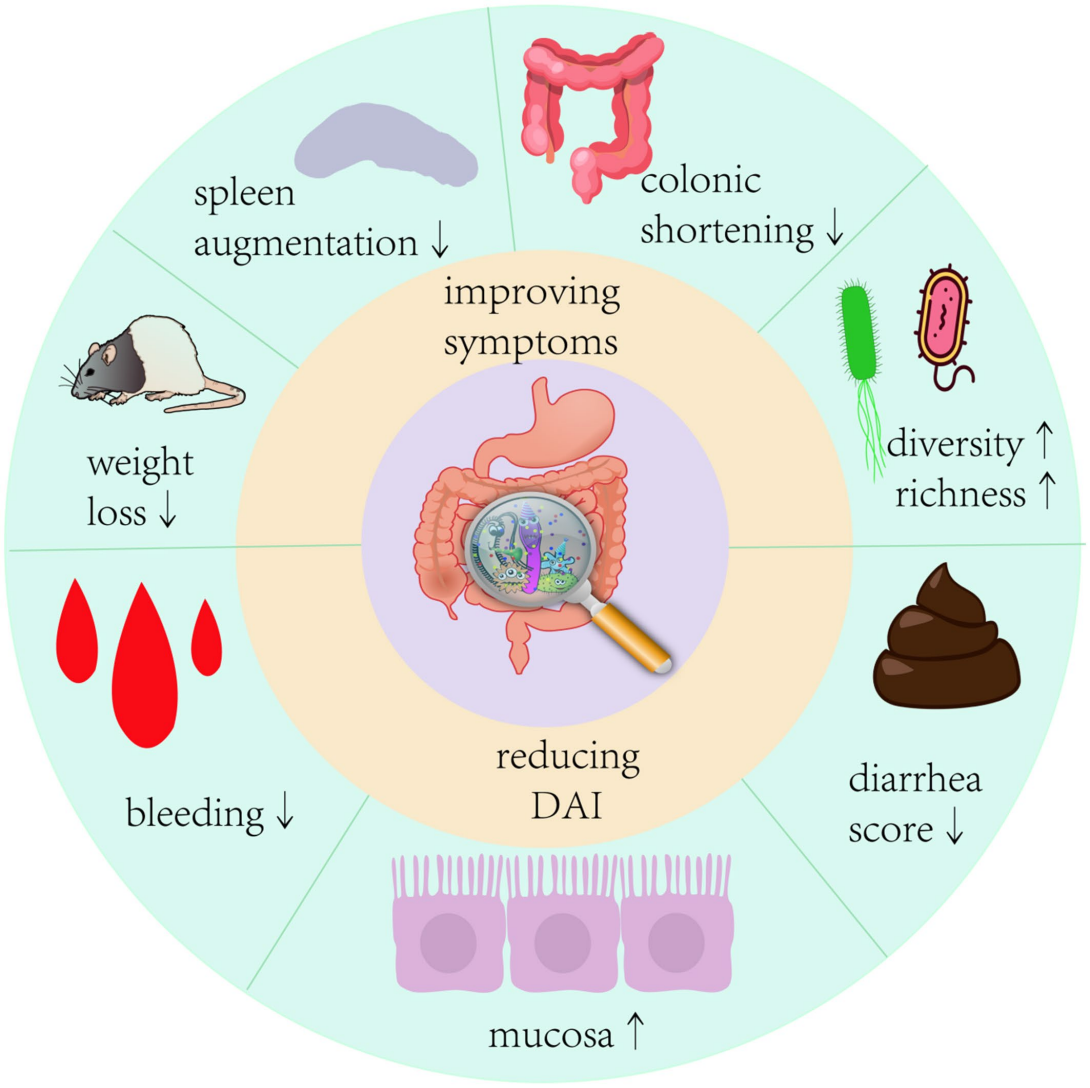
Therapeutic effects of probiotics on colitis in mice Probiotic intervention given to mice with colitis model showed significant improvements in colonic shortening, mucosal damage, weight loss, a decrease in disease activity index, and a significant improvement in the structure of the intestinal flora of the mice.

The International Scientific Association of Probiotics and Prebiotics panel defined probiotics in 2014 as “live microorganisms which when administered in adequate amounts confer a health benefit on the host” (Hill et al., 2014). With a better understanding of the function of the gut microbiota, various probiotics, such as *Bifidobacterium*, *Lactobacillus*, and *Bacillus*, have been proven to be useful to the human body. They colonize the gut and correct the aberrant bacterial composition of the host by increasing the number of symbiotic bacteria (Wieërs et al., 2019). In various investigations, it was shown that probiotics, particularly *Bifidobacterium* and *Lactobacillus*, significantly decreased as the gut microflora in IBD patients became more aberrant (Van der Waal et al., 2019). Pathological signs of UC primarily include epithelial destruction of the colon. Probiotics improve intestinal barrier integrity by metabolizing SCFAs, tryptophan, and other chemicals to increase the production of mucin and tight junction (TJ) proteins in intestinal epithelial cells (Cochrane IBD Group et al., 2020; Lavelle and Sokol, 2020). Recent evidence suggests that probiotics can regulate intestinal immunity, prevent excessive activation of intestinal immune cells, reduce levels of pro-inflammatory factors such as IL-6, INF-γ, TNF-α, and IL-1β, increase levels of anti-inflammatory factors IL-10 and TGF-β, and inhibit the expression of the NF-κB signaling pathway to improve intestinal inflammation (Blacher et al., 2017; Popov et al., 2021). Several modern single-strain probiotics have shown promising results in animal models. A mixed probiotic called VSL#3, which is a combination of eight beneficial bacteria strains and is frequently used in clinical studies, is effective in treating UC in children and adults (Miele et al., 2009; Lee et al., 2012; Oliva et al., 2012). However, some clinical findings imply that probiotics have no significant impact on the maintenance of UC remission (Wildt et al., 2011). Furthermore, while probiotics are effective in preventing the development of acute storage pouchitis and the recurrence of chronic storage pouchitis in adults, research on children with storage pouchitis is lacking. There are different viewpoints on the use of probiotics in the treatment of pediatric UC; some guidelines claim that probiotics are not required, whereas others propose using VSL#3 or *Escherichia coli* Nissle1917 as adjuvant therapy to improve symptoms in pediatric patients with UC (Cheng et al., 2021). Undeniably, different probiotic strains have value in the treatment of UC, but their efficacy and safety require in-depth research due to a paucity of large-scale case–control studies and data on long-term clinical efficacy. In animal model experiments, the application of probiotics had multiple improvements in the symptoms of colitis in mice (Figure 2). Therefore, this review collected animal studies (Tables 1, 2) on the treatment of UC by a variety of potentially beneficial bacteria over the last 5 years, providing a reference for the clinical use of probiotics for the treatment of UC.

**Table 1:** Effects of *Bifidobacterium* on animal models.

Probiotics	Strain	Animal	Interventions	Outcomes	Reference
*Bifidobacterium longum*	YS108R	C57BL/6 J mice *n* = 8	0.2 mL once daily fermented milk for 14 days	Proved to decrease disease active index and MPO activity, decreased the expression of IL-6 and IL-17A and maintained the tight junction proteins, increased the expression of mucin2, modulated the gut microbiota	Yan et al. (2020)
*Bifidobacterium longum*	YS108R	C57BL/6 J mice *n* = 8	5 × 10^9^ *CF*/ mL 0.2 mL noce daily for 14 days	Alleviate the colonic damage, increased the level of IL-10, increased the expression of mucin2 and TJP, maintained gut microbiota imbalance.	Yan et al. (2019)
*Bifidobacterium longum*	51A	BALB/c mice *n* = 14	5 × 10^9^ CFU/mL 0.1 mL once daily for 17 days	Preserved the intestinal architecture, reduced intestinal permeability, and colon injuries.	Abrantes et al. (2020)
*Bifidobacterium longum*	LC67	C57BL/6 mice *n* = 6	1 × 10^9^ CFU once daily for 3 days	Inhibited colon shortening and MPO acitivity, restored disturbance of gut microbiota, improved tight junction protein expression, restored Th17/Treg balance.	Jang et al. (2018)
*Bifidobacterium longum*	HB5502	C57BL/6c mice *n* = 6	4 × 10^9^ CFU/dose once daily for 7 days	Improved intestinal inflammation and fecal microbiota imbalance	Chen et al. (2019)
*Bifidobacterium longum* and Selenium-enriched *Bifidobacterium longum*	DD98	C57BL/6 mice *n* = 6	1 × 10^10^ CFU/kg once daily for 21 days	Decreased the disease severity of UC mice, improved colon lengthened and pathological phenotype, decreased the expression of pro-inflammatory cytokines and oxidative stress parameters, improved the intestinal barrier integrity, promoted the abundance of health-benefiting taxa.	Hu et al. (2022)
*Bifidobacterium longum*	NK151, NK173, and NK175	*Bifidobacterium longum* *n* = 8 or 10	1 × 10^9^ CFU once daily for 5 days	Both suppressed LPS-induced expression of proinflammatory cytokines in macrophages, alleviated Colonic Inflammation.	Yoo et al. (2022)
*Bifidobacterium longum*	CECT 7894	C57BL/6 mice *n* = 6	5 × 10^8^ CFU once daily for 5 days	Decreased weight loss, disease activity index (DAI) scores, colon length shortening, histological damage, increased ZO-1, and Occludin expressions.	Xiao et al. (2022)
*Bifidobacterium breve*	H4-2 and H9-3	C57BL/6 J mice *n* = 8	1 × 10^9^ CFU/mL 0.2 mL once daily for 7 days	Increased the expression of mucin, occludin, claudin-1, ZO-1, decreased the levels of IL-6, TNF-α, IL-1β and increased IL-10, inhibited the expression of the NF-κB signaling pathway, increased the levels of SCFAs, reduced the abundance of Proteobacteria and Bacteroidea, and increased the abundance of Muribaculaceae.	Niu et al. (2022)
*Bifidobacterium breve*	CCFM683 and BJCP1M6	C57BL/6 J mice *n* = 8	5 × 10^9^ CFU/mL 0.2 mL once daily for 14 days	Alleviated the inflammation, increased the concentration of mucin2 (MUC2) and goblet cells, up-regulated the tight junction (TJ) proteins and ameliorated the epithelial apoptosis, rebalanced the damaged gut microbiota.	Chen et al. (2021)
*Bifidobacterium lactis*	A6	C57BL/6 J mice *n* = 8	4 × 10^9^ CFU once daily for 21 days	Inhibited DSS-induced bodyweight loss and colon shortening, improved intestinal barrier integrity, attenuated the oxidative stress, downregulated TNF-α, IL-1β and IL-6 levels and upregulated IL-10 level.	Wang et al. (2022)
*Bifidobacterium lactis*	BB12	C57BL/6 J mice *n* = unknown	1.2 × 10^10^ CFU twice a day for 7 days	Ameliorated DSS-induced colitis, reduced tumor necrosis factor-α-mediated IEC apoptosis	Chae et al. (2018)
*Bifidobacterium lactis*	5,764	C57BL/6 J or BALB/c mice	5 × 10^8^ CFU once daily for 5 days	Alleviated inflammatory responses.	Hrdý et al. (2020)
*Bifidobacterium bifidum*	FJSWX19M5	BALB/c mice *n* = 10	5 × 10^9^ CFU/mL 0.2 mL once daily for 5 weeks	Restored the disruption of the gut microbiota, increased IL-10 levels, alleviated body weight loss, colonic shortening and injury.	Qu et al. (2023)
*Bifidobacterium bifidum*	FL-276.1 and FL-228.1	BALB/c mice *n* = 10	1 × 10^9^ CFU/mL 0.1 mL once daily for 22 days	Ameliorated colitis symptoms, improved the intestinal barrier integrity, decreased the levels of IL-6, TNF-α.	Cui et al. (2022)
*Bifidobacterium bifidum*	WBIN03	BALB/c mice *n* = 10	1 × 10^9^ CFU/mL 0.3 mL once daily for 4 days.	Regulated the colitis gut microbiota, protected the mucosal barrier system, improved the antioxidant levels, decreased the weight loss and DAI, reduced the expression of TNF-α, up-regulated the expressions of IL-10.	Wang et al. (2018)
*Bifidobacterium infantis*	ATCC 15697	C57BL/6 mice	2 × 10^8^ CFU/mL 0.2 mL once daily for 21 days	Alleviated colitis symptoms, alleviated inflammatory cell infiltration, improved oxidative stress, reduced the colonic inflammatory cytokine levels.	Sheng et al. (2020)
*Bifidobacterium infantis*	FJSYZ1M3	C57BL/6 N mice *n* = 8	1 × 10^10^ CFU/mL 0.2 mL once daily for 14 days	Improved colitis symptoms, increased the concentration of tight junction proteins, reduced the levels of pro-inflammatory cytokines, enlarged the species richness of gut microbiota	Li et al. (2023)
*Bifidobacterium adolescentis*	NK98	C57BL/6 mice *n* = 6	1 × 10^9^ CFU once daily for 5 days	Reduced colon shortening, colonic myeloperoxidase activity, proinflammatory cytokine IL-6 and IL-1β expression, and NF-κB activation.	Jang et al. (2019)
*Bifidobacterium adolescentis*	Reuter 1963	BALB/c mice *n* = 16	1 × 10^9^ CFU/mL for 7 days	Enlarged the species richness of gut microbiota, improved colitis symptoms.	Ghadimi et al. (2021)
*Bifidobacterium adolescentis*	ATCC15703	BALB/c mice *n* = 6	1 × 10^9^ CFU/mL o.3 mL once daily for 21 days	Decreased diarrhea score, spleen weight, and increased colon length.	Fan et al. (2021)
*Bifidobacterium adolescentis*	IF1-11 and IF1-03	C57BL/6 mice *n* = 5	5 × 10^8^ CFU once daily for 15 days	Decreased the levels of IL-6, TNF-α, increased IL-10 levels, induced abundant Th17 cells.	Yu et al. (2019)

**Table 2:** Effects of *Lactobacillus* on animal models.

Probiotics	Strain	Animal	Interventions	Outcomes	Reference
*Lactobacillus plantarum*	AR17-1	C57BL/6 mice *n* = 16	5 × 10^8^ CFU once daily for 10 days	Reduce diarrhea, reduced the DAI score, prevented colon shortening, decreased MPO activity, reduced the expression of TNF-α.	Wang et al. (2021)
*Lactobacillus plantarum*	Q7	C57BL/6 J mice *n* = unknown	0.5-1 mg /kg once daily for 10 days	Improved the shortening of the colon and weight loss, reduced the spleen index, improved colon damage, decreased the expression of inflammatory cytokines, restore the gut microbiota.	Hao et al. (2021)
*Lactobacillus plantarum*	Y44	BALB/c mice *n* = 10	1 × 10^8^ CFU/mL-1 × 10^9^ CFU/mL0.2 mL once daily for 6 weeks	Restored crypt structure and increased goblet cells, reversed the declines in the SOD, GPx, and CAT activities, activate the hepatic Nrf-2/Keap-1 pathway, reversed the downregulation of claudin-1 and occludin protein expressions, altered the diversity of gut microbiota	Gao et al. (2021)
*Lactobacillus plantarum*	12	BALB/c mice *n* = 10	1 × 10^7^ CFU/mL and 1 × 10^9^ CFU/mL 0.3 mL once daily for 7 days	Restored gut microbiota, enforced the intestinal barrier function, ameliorated intestinal inflammation.	Sun et al. (2020)
*Lactobacillus plantarum*	N13 and CCFM8610	BALB/c mice *n* = 10	3 × 10^9^ CFU/0.2 mL once daily for 7 days	Decreased body weight, DAI score, and colon length shorting, increased gut microbiota diversity, decreased Pro-inflammatory cytokine expression, remediated colon damage.	Liu et al. (2020)
*Lactobacillus plantarum*	CAU1055	ICR mice *n* = 8	4 × 10^10^ cfu/mL 0.2 mL once daily for 3 weeks	Inhibited body weight loss, colon shortening, and colon damage, reduced levels of INF-α, IL-6.	Choi et al. (2019)
*Lactobacillus plantarum*	L15	C57BL/6 mice *n* = 12	1 × 10^10^ CFU /ml 0.2 mL/once daily for 21 days	Reducted pro-inflammatory cytokine and increased anti-inflammatory cytokine, protected epithelial integrity, improved LPS and D-lactic acid concentrations, reshaped the gut microbiota structure, regulated SCFAs production, suppressed NF-κB pathway	Wang et al. (2022)
*Lactobacillus plantarum*	HNU082	C57BL/6 J mice *n* = 12	1 × 10^9^ CFU/mL 0.2 mL once daily for 7 days	Optimized the species composition and the structure, increased the levels of SCFAs, goblet cells, mucin2, claudin-1 and claudin-2, ZO-1, IL-10, TGF-β1, and TGF-β2, decreased IL-6, TNF-α and MPO.	Wu et al. (2022a)
*Lactobacillus plantarum*	HNU082	C57BL/6 J mice *n* = 8	1 × 10^9^ CFU/mL for 7 days	Increased body weight and colon length, decreased DAI, immune organ index, inflammatory factors, and histopathological scores, improved the intestinal mucosal barrier,.	Wu et al. (2022b)
*Lactobacillus plantarum*	ZS62	C57BL/6 mice *n* = 10	10 × 10^9^ CFU/mL 0.1 mL/g once daily for 4 weeks	Inhibited atrophy of the mouse colon, reduced the histopathological damage, enhanced the antioxidant capacity, reduced the release of proinflammatory cytokines,	Pan et al. (2021)
*Lactobacillus plantarum*	06 cc2	C57BL/6 mice *n* = 12	20 mg twice a day for 20 days	Increased levels of IL-10, improved weight loss,.	Tanaka et al. (2020)
*Lactobacillus plantarum*	CBT LP3	C57BL/6 mice *n* = 15	1 × 10^8^ CFU once daily for 7 days	Induced weight loss and DAI scores, reduced inflammatory infiltrates, restored intestinal epithelia, suppressed expression of proinflammatory cytokines.	Kim et al. (2020)
*Lactobacillus plantarum*	MTCC 5690	Swiss Albino mice *n* = 8	1 × 10^9^ CFU/mL 0.2 mL once daily for 14 days	Improved intestinal permeability, decreased MPO activity, improved health index and better growth.	Pradhan et al. (2019)
*Lactobacillus plantarum*	YS3	C57BL/6 J mice *n* = 10	1 × 10^9^ CFU/mL and 1 × 10^8^ CFU/mL 0.2 mL once daily for 5 weeks	Decreased levels of MDA, MPO, and NO, and increased the levels of GSH, increased levels of IL-2, decreased levels of IL-6, improved colonic tissue damage.	Hu et al. (2021)
*Lactobacillus plantarum*	CQPC06	C57BL/6 J mice *n* = 10	1 × 10^9^ CFU/mL and 1 × 10^8^ CFU/mL 0.2 mL once daily for 7 days	Improved body weight loss, colon length, reduced the leves of proinflammatory cytokines and goblet cells, decreased the levels of MPO and NO.	Zhang et al. (2018)
*Lactobacillus plantarum*	QS6-1、QHLJZD20L2 and VJLHD16L1	C57BL/6 J mice *n* = unknown	0.2 mL once daily for 7 days	Increased the expression of ZO-1 and occluding, recovered gut barrier integrity, reduced weight loss, increased colon length.	Liu et al. (2022)
*Lactobacillus plantarum*	AR326	IRC mice *n* = 5	2 × 10^9^ CFU/mL 0.2 mL once daily for 7 days	Restored the tight junction protein expression and reduced of the pro-inflammatory cytokines, decreased the weight loss, DAI, colon length shortening, MPO activity, and colon epithelial damage.	Wang et al. (2019)
*Lactobacillus plantarum*	CCFM242	C57BL/6 mice *n* = 5	2 × 10^9^ CFU once daily for 7 days	Reduced the levels of IL-1β and IL-6, and enhanced the level of IL-10, decreased the weight loss, colon length shortening, MPO activity, and colon barrier.	Zhai et al. (2019)
*Lactobacillus plantarum*	AB-1 and SS-128	C57BL/6 mice *n* = 6	2 × 10^9^ CFU/mL 0.2 mL once daily for 7 days	Decreased the weight loss, DAI, MPO activity and colon length shortening, increased mucosal integrity, increased inflammatory cytokine expression, enreached diversity of the gut microbiota.	Qian et al. (2022)
*Lactobacillus plantarum*	IMAU10216, IMAU70095 and IMAU 10,120	C57BL/6 mice *n* = 4	1 × 10^10^ CFU/mL 0.1 mL once daily for 21 days	Decreased the weight loss, improved colonic tissue damage, increased goblet cell, alleviated the MPO accumulation, decreased colon cytokine levels, increased microbial diversity.	Khan et al. (2022)
*Lactobacillus plantarum*	ZDY2013	BALB/c mice *n* = 10	1 × 10^9^ CFU/mL 0.3 mL once daily for 4 days	Regulated the colitis i gut microbiota, protected the mucosal barrier system, improved the antioxidant levels, decreased the weight loss and DAI, reduced the expression of TNF-α, up-regulated the expressions of IL-10.	Wang et al. (2018)
*Lactobacillus plantarum*	LC27	C57BL/6 mice *n* = 6	1 × 10^9^ CFU once daily for 3 days	Inhibited colon shortening and MPOacitivity, restored disturbance of gut microbiota, improved tight junction protein expression, restored Th17/Treg balance.	Jang et al. (2018)
*Lactobacillus rhamnosus*	M9	C57BL/6NCrSlc mice *n* = 10	2 × 10^9^ CFU once daily for 14 days	Upregulated the fecal microbial diversity and reversed fecal microbial functions, ameliorated inflammation.	Xu et al. (2021)
*Lactobacillus rhamnosus*	MTCC-5897	albino weanling mice *n* = 9	2 × 10^9^ CFU/ mL once daily for 28 days	Reduced the DAI, diminished levels of pro-inflammatory and enhanced levels of the anti-inflammatory cytokine, improved immune homeostasis and intestinal barrier integrity.	Kaur et al. (2021)
*Lactobacillus rhamnosus*	LDTM 7511	C57BL/6 J mice *n* = 8	1 × 10^9^ CFU once daily for 14 days	Alleviated the release of inflammatory, induced the transition of gut microbiota from dysbiotic conditions.	Yeo et al. (2020)
*Lactobacillus rhamnosus*	SHA113	C57BL/6Cnc mice *n* = 10	1 × 10^9^ CFU/mL once daily for 10 days	Reduced body weight loss, colon length shorting, and DAI, improved colon structural integrity.	Pang et al. (2021)
*Lactobacillus rhamnosus*	GG	BALB/c mice *n* = unknown	1 × 10^9^ CFU/ ml every other day for 3 weeks	Decreased DAI score, prevented colon shortening, maintained mucosal integrity, decreasedpro-inflammatory cytokines, improved gut microbiota.	Son et al. (2019)
*Lactobacillus rhamnosus*	GG	C57BL/6 mice *n* = 8	1 × 10^6^ CFU for 8 days	Prevented colon shortening, decreasedpro-inflammatory cytokines.	Jia et al. (2020)
*Lactobacillus rhamnosus*	1.0320	C57BL/6 mice *n* = 13	2 × 10^8^ CFU/0.2 mL every other day for 28 days	Reduced DAI score, decreased MPO activity, increase hemoglobin content, regulate the expression levels of inflammatory cytokines, increase the abundance and diversity of gut microbiota.	Liu et al. (2020)
*Lactobacillus rhamnosus*	KLDS 1.0386	C57BL/6 J mice *n* = 8	1 × 10^9^ CFU/mL for 21 days	Decreased DAI score, MPO level, and pro-inflammatory cytokines, increased anti-inflammatory cytokine, tight junction proteins and mucins.	Shi et al. (2020)
*Lactobacillus acidophilus*	ATCC4356	SD rat *n* = 10	1 × 10^8^ CFU/0.5 mL once daily for 8 days	Meliorated colitis symptoms, recovered of the colon barrier, contributed to the balance of inflammation and oxidative stress.	Li et al. (2022)
*Lactobacillus acidophilus*	BIO5768	BALB/c mice *n* = 6	5 × 10^8^ CFU once daily for 5 days	Triggers IL-17-dependent innate defense response, activation of innate lymphoid cells type 3 and improves colitis.	Hrdý et al. (2022)
*Lactobacillus acidophilus*	KBL402 and KBL409	C57BL/6 J mice *n* = 8	1 × 10^9^ CFU once daily for 8 days	Both improved colitis symptoms, downregulated Th1-, 2- and 17-related cytokines, decreased levels of MPO, improved cecal microbiota.	Kim et al. (2021)
*Lactobacillus acidophilus*	XY27	C57BL/6 mice *n* = 8	1 × 10^9^ CFU/mL 0.1 mL/10 g for 3 weeks	Decreased DAI score, prevented colon shortening, decreased levels of oxidative stress and inflammatory cytokines.	Hu et al. (2020)
*Lactobacillus acidophilus*	CCFM137, FAHWH11L56, FGSYC48L79,	C57BL/6 mice *n* = 8	1 × 10^9^ CFU/mL 0.2 mL once daily for 14 days	CCFM137 and FAHWH11L56 showed potential for relieving colitis, FGSYC48L79 exacerbated colitis	Huang et al. (2022)
*Lactobacillus acidophilus*	CGMCC 7282	SD rats *n* = 10	1 × 10^9^ CFU once daily for 14 days	Reduced in the colon/body weight ratio and the colon weight/length ratio, recoved intestinal structure, increased ZO-1 expression, reduction of inflammatory cytokines, recovered gut microbiota.	Kaur et al. (2020)
*Lactobacillus reuteri*	R28	C57BL/6 mice *n* = 16	5 × 10^8^ CFU once daily for 10 days	Reduce diarrhea, reduced the DAI score, prevented colon shortening, decreased MPO activity, reduced the expression of TNF-α.	Wang et al. (2021)
*Lactobacillus reuteri*	R2LC and ATCC PTA 4659	C57Bl/6 mice	1 × 10^8^ CFU once daily for 7 days	Reduced DAI score, reduced histological signs of tissue damage, reduced levels of proinflammatory cytokines, increased the thickness of the colonic firmly adherent mucus layer, upregulates the expression of tight junction proteins.	Ahl et al. (2016)
*Lactobacillus reuteri*	ATCC PTA 4659	C57BL/6 J mice *n* = 6–8	1 × 10^8^ CFU once daily for 7 days	Reduced weight loss, DAI score, and colon shortening, preserved the microbiota diversity, induced the expression of tight junction proteins and intestinal heat shock proteins, increased the ileal villus height.	Liu et al. (2022)
*Lactobacillus reuteri*	DSM 17938	C57BL/6 J mice *n* = 9	1 × 10^7^ CFU once daily for 14 days	Boosted treg cells in the intestinal mucosa, increase in alpha diversity, increased tryptophan metabolism.	Liu et al. (2019)
*Lactobacillus reuteri*	R2LC	C57Bl/6 mice *n* = 9	1 × 10^8^ CFU once daily for 14 days	Reduced weight loss, DAI, and colon shortening, preserved the microbiota diversity.	Liu et al. (2021)
*Lactobacillus gasseri*	G098	C57BL/6 J mice *n* = 8	4 × 10^9^ CFU/mL 0.2 mL once daily for 10 days	Alleviated Inflammatory manifestations, reversed Changes in serum pro−/anti-Inflammatory cytokine levels, improved gut microbiota diversity, increased metabolic pathways	Zhang et al. (2022)
*Lactobacillus paracasei*	NTU 101	C57BL/6 mice *n* = 10	2.3× 10^9^ CFU/kg once daily and 4.5 × 10^9^ CFU/kg once daily for 25 days	Improved anti-oxidant capacity, reduced pro-inflammatory cytokine levels, increased anti-inflammatory cytokine levels, and ameliorated body weight loss	Chen et al. (2019)
*Lactobacillus paracasei*	R3	C57BL/6 mice *n* = 6	1 × 10^9^ CFU/mL 0.2 mL once daily for 14 days	Reduced DAI scores, alleviated colon injury, regulated Th17/Treg cell balance.	Huang et al. (2021)
*Lactobacillus paracasei*	N1115	C57BL/6 mice *n* = 14	1 × 10^7^ CFU once daily from postnatal day 1 to day 7 and 10^8^ CFU once daily from day 8 to day 14	Decreased severity of intestinal tissue injury, cell apoptosis, and proinflammatory cytokines expression	Xun et al. (2022)
*Lactobacillus johnsonii*	similarity of 99% to UMNLJ22	C57BL/6 mice *n* = 12	1 × 10^9^ CFU every other day(7 times)	Normalized colon length and spleen weight, attenuated colonic hyperplasia, suppressed the secretion of inflammatory cytokines and infiltration of immune cells, restored the abnormal expression of antimicrobial peptides, attenuated ER stress–related cell death.	Zhang et al. (2021)
*Lactobacillus kefiranofaciens*	JKSP109	C57BL/6 mice *n* = 1	2 × 10^8^ CFU/mL 0.2 mL once daily for five days per week for 17 weeks	Recovered of the colon barrier, modified gut microbiota, decreased the levels of proinflammatory cytokines, increased SCFA concentrations	Wang et al. (2022)
*Lactobacillus curvatus*	BYB3	C57BL/6 mice *n* = 6	1 × 10^9^ CFU/mL 0.2 mL once daily for 14 days	Alleviated the disruption of Intestinal barrier function, alleviated colitis symptoms, Inhibiting the Production of IL-6, TNF-R1, TNF-R2, and TNF-α.	Dong et al. (2022)

### 2.1. Effects of *Bifidobacterium* spp. on UC

The genus *Bifidobacterium* belongs to the gram-positive bacteria of the phylum Actinobacteria and is one of the first microorganisms to colonize the intestine (O’Callaghan and Van Sinderen, 2016). *Bifidobacterium* is also immunotolerant in humans and not subject to rejection (Yao et al., 2021). Hence, several species of this genus constitute the main group of probiotics (Fontana et al., 2013; Mianzhi and Shah, 2017). Exopolysaccharides (EPS), SCFAs, and conjugated linoleic acid (CLA), which are produced during metabolism, act on intestinal epithelial cells and play a role in regulating intestinal homeostasis (Hughes et al., 2017) by improving intestinal barrier function and modulating the intestinal mucosal immune system and inflammatory response (Dodd et al., 2017). Numerous animal studies have shown that *Bifidobacterium* can increase the population of the dominant flora, which is absent in UC, and ameliorate symptoms of dextran sulfate sodium (DSS)-induced colitis. Furthermore, conventional medications mixed with probiotics can enhance therapeutic efficacy and improve the remission rate in UC. However, because each *Bifidobacterium* strain attenuates the inflammatory response differently and responds differently to cytokines (Choi et al., 2022), we believe that the function of probiotics should be subdivided based on strain characteristics. When considering the status of patients to achieve the optimal therapeutic effect, the appropriate probiotic should be selected.

#### 2.1.1. *Bifidobacterium longum* alleviates oxidative stress

*Bifidobacterium longum* is one of the most prevalent bacteria in the intestine. The main metabolites of *B. longum* are SCFAs and CLA (Albert et al., 2019), which alleviate colonic inflammation by increasing antioxidant activity and regulating the production of reactive oxygen species, leading to reduced oxidative stress (Yao et al., 2021). Numerous animal studies have shown that *B. longum* can improve experimental colitis. *B. longum* strains YS108R, 51A, and LC67 can attenuate DSS and 2,4,6-trinitrobenzene sulfonic acid (TNBS)-induced colonic damage by strengthening the mucosal barrier and regulating the composition of the gut microbiota (Jang et al., 2018; Yan et al., 2019; Abrantes et al., 2020; Yan et al., 2020; Bi et al., 2022). Furthermore, mice in the *B. longum* CCFM681 intervention group outperformed those in the CCFM760 and CCFM642 groups in terms of the improvement in mucosal barrier function and reduction of mucosal barrier levels, with the results of the analysis of bacterial products revealing that the improvements were related to the amount of CLA produced by *B. longum*; higher CLA levels were associated with greater colitis remission (Chen et al., 2021). In cell experiments, *B. longum* strains CCFM752, CCFM1149, CCFM10, and LTBL16 attenuated intestinal inflammatory responses by increasing intracellular catalase activity, lowering NADPH oxidase activation, and improving intracellular antioxidant capacity (Huang et al., 2020; Yan et al., 2020; Wang et al., 2021; Yao et al., 2021). Notably, ESP produced by *B. longum* 35,624 induced the secretion of relatively low levels of cytokines from human dendritic cells and attenuated the accumulation of IL-17 in the intestine (Schiavi et al., 2016). Similarly, *B. longum* YS108R produces ropy exopolysaccharides that suppress the immunological response, reducing intestinal inflammation (Yan et al., 2020). Additionally, Chen (Chen et al., 2019) investigated the effects of similar doses (4 × 10^9^ CFU/dose) of *B. longum* HB5502 and VSL#3 on colitis in mice. The study found that both the two strains relieved colonic inflammation, reduced serum inflammatory factors, and increased tight ligin expression, demonstrating that there is no significant difference in the results between a single strain of *B. longum* HB5502 and a mixed probiotic containing *B. longum*. However, whether this suggests that *B. longum* plays a major role in probiotics requires further investigation. Furthermore, another selenium-rich strain of *B. longum*, namely DD98, ameliorates UC-induced selenium deficiency by producing selenoproteins, which also possess substantial anti-inflammatory and immunomodulatory activities to improve DSS-induced intestinal inflammation in mice (Hu et al., 2022). Additionally, *B. longum* strains NK173, NK151, and NK175 alleviate stress fatigue, depression, and symptoms of UC by regulating the expression ratio of pro- and anti-inflammatory cytokines and gut microbiota byproducts (e.g., lipopolysaccharide, LPS) (Yoo et al., 2022), making them potential probiotics for the treatment of patients with UC who also have neuropsychiatric disorders. Meanwhile, the combination of *B. longum* CECT 7894 and infliximab improved the therapeutic effect of both drugs, with greater improvement of symptoms in the *B. longum* group compared to the infliximab-only group (Xiao et al., 2022). A prior clinical trial found that combining *B. longum* trisporus with mesalazine improved the therapeutic effect of drugs used for UC (Jiang et al., 2020), inferring that trisporus could be a safe and convenient supplementary treatment option for patients who are resistant to traditional medications. Overall, *B. longum* may be a useful adjuvant biologic drug in the treatment of UC (Li et al., 2023).

#### 2.1.2. *Bifidobacterium breve* enhances the intestinal mucosal barrier and reduces levels of inflammatory factors

*Bifidobacterium breve* is a non-budding, non-motile, gram-positive, specialist anaerobic bacteria. Like *B. longum*, *B. breve* can lower the intestinal inflammatory response via EPS. Moreover, the improvement was greater with *B. breve* H4-2 than with H9-3, which may be related to the higher production of EPS by H4-2 (Niu et al., 2022). Similarly, *B. breve* strains CCFM683 and BJCP1M6B reduced the levels of TNF-α and IL-6, significantly increased the levels of mucin-2 (MUC2) and cupped cells, upregulated the expression of TJ proteins, and improved DSS-induced epithelial cell apoptosis; in contrast, neither *B. breve* strains FHLJDQ3M5 nor M2CF22M7 showed these effects (Chen et al., 2021). *B. breve* YH68 reduced the population of *C. difficile* and toxin levels in feces in an animal study, and *B. breve* CCFM1025 improved symptoms of major depression by modulating tryptophan metabolism in a randomized controlled trial (Yang et al., 2022), providing another therapeutic option for patients with UC with concomitant symptoms of depression. However, the role of *B. breve* in maintaining UC remission was not significant, and there was no significant difference in relapse-free survival between the *B. breve* and placebo groups after 48 weeks of oral administration of fermented yogurt containing primarily *B. breve* (Matsuoka et al., 2018). The results contradict those of a prior animal study, and the difference may be related to the probiotic dose, delivery route, and probiotic production process. However, this does not rule out the beneficial effects of *B. breve* for the treatment of UC.

#### 2.1.3. *Bifidobacterium animalis* subsp. *lactis* has potential as a supplement for the treatment of UC due to its anti-inflammatory properties

*Bifidobacterium lactis* is a gram-positive anaerobic bacterium found in the intestines of most animals. *B. lactis* adheres to the epithelial mucosa in vast numbers and is an important component of a healthy gut microbiota (Masco et al., 2004). Regarding the efficacy of *B. lactis* on UC, mice with DSS- or TNBS-induced colitis treated with *B. lactis* strains A6, BB12, and 5,764 showed significant improvements in intestinal barrier function and immunomodulation (Chae et al., 2018; Hrdý et al., 2020; Wang et al., 2022). Furthermore, *B. lactis* exerts its anti-inflammatory properties through the activation of peripheral blood mononuclear cells and increasing forkhead box P3 (FOXP3) gene expression to increase TGF-β levels (Shakurnia et al., 2019). Notably, *B. lactis* BL-99 not only reduced intestinal inflammation in UC but also alleviated colitis-related lung injury by altering SCFA production and inflammatory cell ratios (Nan et al., 2023). Preliminary evidence suggests that *B. lactis* is another potentialz option for reducing inflammation in UC as well as improving extraintestinal manifestations in patients with UC.

#### 2.1.4. *Bifidobacterium bifidum* supplementation is effective in treating UC

*Bifidobacterium bifidum* is the dominant gram-positive anaerobic bacterium in the human intestine and plays a significant role in the prevention of gastrointestinal dysfunction (Andresen et al., 2020). Animal experiments have suggested that the role of *B. bifidum* FJSWX19M5 in improving TNBS-induced chronic colitis is achieved through repairing gut barrier damage and enhancing Tregs (Qu et al., 2023). *B. bifidum* strains FL-276.1 and FL-228.1 improve immune function by activating the aryl hydrocarbon receptor (AHR) (Cui et al., 2022). Additionally, improvement in the symptoms of colitis brought about by *B. bifidum* B1628 was accompanied by obvious gut microbiota remodulation (Feng et al., 2022). Furthermore, *B. bifidum* BGN4-SK alleviated DSS-induced colitis by producing antioxidant enzymes and reducing pro-inflammatory cytokine production. However, *B. bifidum* BGN4-pBESIL10 had little effect on IL-10 production and the improvement of colitis (Kang et al., 2022). Interestingly, *B. bifidum* improved the TJ barrier function in a strain-specific manner, with *B. bifidum* BB1 demonstrating the greatest improvement (Al-Sadi et al., 2021a). Furthermore, heat-inactivated and lysozyme-treated *B. bifidum* strains FJSWX19M5 and BGN4 also improved symptoms of colitis, but the degree of improvement was not similar to that of live bacteria. However, lysozyme-treated *B. bifidum* BGN4 maintained the intestinal mucosal barrier function better than live bacteria (Lee et al., 2022; Qu et al., 2023), which may be related to the stronger adhesion of the inactivated strain. In summary, *B. bifidum* is a potent probiotic that can improve colitis in a variety of ways, including by regulating gut microbiota, improving intestinal barrier function, and modulating immunity; however, the effects of *B. bifidum* are affected by the type of strain and organism. Nevertheless, *B. bifidum* is a promising probiotic that should undergo further investigation for its specific therapeutic properties.

#### 2.1.5. *Bifidobacterium adolescentis* treats UC by balancing the gut microbiota

As a gram-positive anaerobic bacterium, *B. adolescentis* is the dominant bacterium in the intestinal tract of young people and plays an important role in the treatment of constipation, anxiety, depression, and colitis (Jang et al., 2019). Current research shows that *B. adolescentis* Reuter 1963 can reverse dysbiosis of the gut microbiota caused by peptidoglycan recognition protein 3 deficiency (Ghadimi et al., 2021). Oral administration of *B. adolescentis* ATCC15703 relieves symptoms of chronic colitis in mice by regulating the immune response, making it a potential probiotic (Fan et al., 2021). *B. adolescentis* IF1-03 with high-molecular-weight EPSs can activate dendritic cells or macrophages, and both rely on the toll-like receptor 2-ERK/p38 MAPK signaling cascade to skew Treg/Th17 cells to protect mice from DSS-induced colitis (Yu et al., 2019). *B. adolescentis* has an obvious antioxidant effect, but the SOD level in the intestinal tract of model mice was not measured in the above experiments. Hence, the effects of antioxidants on improving colitis could not be evaluated. Future studies should further explore whether *B. adolescentis* has other properties that may improve UC.

#### 2.1.6. *Bifidobacterium infantis* alleviates symptoms of UC by reducing the inflam-matory response

*Bifidobacterium infantis* is the dominant bacteria in breastfed infants; thus, it has a high abundance in infancy (Mattarelli et al., 2008). It is acid- and bile-resistant, is strongly adhesive, does not destroy mucus, is non-invasive, and does not harm the intestinal mucosa (Ewaschuk et al., 2008). *B. infantis* FJSYZ1M3 can remarkably reduce the DAI, limit weight loss and colonic shortening via alteration of the gut microbiota, maintain the integrity of the intestinal barrier, and modulate levels of inflammatory cytokines (Li et al., 2023). *B. infantis* also enhances the development of cluster of differentiation (CD) 4+ T cells into Tregs and also increases the expression of IL-10 and TGF-β1, subsequently reducing the inflammatory response in the gut (Zhou et al., 2022). Additionally, co-administration of *B. infantis* ATCC 15697 with xylooligosaccharide demonstrated additional efficacy in protecting against colonic damage due to DSS-induced colitis in mice (Sheng et al., 2020). Moreover, *B. infantis* can enhance the development of the host immune system and maintain elevated IgA and IgG titers in infancy and at 2 years (Huda et al., 2019). Significantly lower levels of pro-inflammatory cytokines on postnatal days 40 and 60 in EVC001-fed infants have been reported, providing a new strategy to improve intestinal inflammation during developmental stages (Henrick et al., 2019). *B. infantis* also exerts anti-inflammatory effects through various pathways. However, the effectiveness of *B. infantis* in the treatment of UC in children remains unclear. *B. infantis* is abundant in the infant gut and may play a major role in inhibiting the development of UC in this population; hence, its effects need to be further explored.

### 2.2. Effects of *Lactobacillus* spp. on UC

*Lactobacillus* is a genus of gram-positive microbes in fermented foods that colonize the human digestive tract in vast numbers; they are also the first probiotics (De Melo Pereira et al., 2018). *Lactobacillus* is distinguished by its capacity to convert glucose, lactose, and galactose into lactic acid, which reduces the intestinal pH, and produce bacteriocins, hydrogen peroxide, and diacetyl to inhibit the growth of other bacteria. *Lactobacillus* has been extensively studied, and its use in the treatment of UC has been recognized (Garbacz, 2022). SCFAs, bacteriocins, and EPS of *Lactobacillus* can exert powerful immunomodulatory effects, and its cell wall can attenuate inflammation and oxidative interference and enhance antioxidant defense (Chorawala et al., 2021). Additionally, *Lactobacillus* can increase levels of catalase (CAT) and superoxide dismutase (SOD) while decreasing those of reactive oxygen species (ROS) at the genetic level (Jin et al., 2020), thus reducing intestinal oxidative stress. *Lactobacillus* is the most extensively utilized genus of probiotic products, and it has been successfully used clinically in the adjuvant treatment of patients with UC.

#### 2.2.1. *Lactobacillus plantarum* has strain specificity in the treatment of UC

*Lactobacillus plantarum* is the most common flora in the gut and is a parthenogenic anaerobic bacterium found in vegetables and fermented fruit juices. *L. plantarum* can supplement human vitamin B by synthesizing folic acid, increasing the stability and absorption of B1, B6, and B12 in the intestine, and secreting antibacterial active peptides to inhibit the growth of many gram-positive bacteria (Yin et al., 2018). *L. plantarum* is a probiotic used in the treatment of UC; in animal studies, oral administration of several *L. plantarum* strains, including AR17-1, Q7, Y44, L15, 12, N13, CCFM8610, CAU1055, HNU082, ZS62, ZDY2013, LC27, 06CC2, CBT LP3, and MTCC 5690, had a preventive effect on the development of UC (Jang et al., 2018; Wang et al., 2018, 2021, 2022; Zhou et al., 2018; Choi et al., 2019; Pradhan et al., 2019; Kim et al., 2020; Liu et al., 2020; Tanaka et al., 2020; Yu et al., 2020; Gao et al., 2021; Hao et al., 2021; Pan et al., 2021; Wu et al., 2022a,b). Additionally, a high concentration (1 × 10^9^ CFU/mL) of *L. plantarum* strains YS3 and CQPC06 had superior effects compared to a low concentration (1 × 10^8^ CFU/mL) of the same strains (Zhang et al., 2018; Shi et al., 2020), indicating that the effect of probiotics may be dose-dependent. Studies have shown that the efficacy of *L. plantarum* is strain-specific (Liu et al., 2022). For example, *L. plantarum* strains CAU1055, which has a strong CLA synthesis ability; AR326, which has strong adhesion; and CCFM242, which is rich in zinc, better relieve symptoms of colitis than other strains (Choi et al., 2019; Wang et al., 2019; Zhai et al., 2019). Meanwhile, *L. plantarum* strains NCIMB8826 and LM0419, which are unable to synthesize bacteriocins, showed no protective effect on mice with TNB-induced colitis (Yin et al., 2018). In addition, EPSs plays an important role in improving the intestinal barrier and gut microbiota. For example, *L. plantarum* NCU116 EPSs regulate colonic epithelial regeneration, and *L. plantarum* YW11 enhances the amount of SCFAs. However, the relevant mechanisms remain obscure but may relate to stimulating the signal transducer and activator of transcription 3 (STAT3) signaling pathway (Yunyun Jiang, 2018; Zhou et al., 2021; Liu et al., 2022). These characteristics may provide guidance in determining the beneficial strains that may be used to produce probiotics. In addition to differences between strains, different forms of the same strain have different efficacy in treating colitis. Studies have found that the AB-1 and SS-128 strains with the autoinducer-2 defect can reduce colon inflammation more significantly than wild-type strains (Qian et al., 2022). Additionally, intervention with *L. plantarum* strains C2 (IMAU10216) and C3 (IMAU70095) 21 days before the induction of colitis in an animal model resulted in less severe symptoms, indicating a preventive effect of *L. plantarum* on colitis development (Khan et al., 2022). *L. plantarum* has been extensively studied at the phenotypic, molecular, and genetic levels, and the results have proven that *L. plantarum* is a powerful and promising probiotic. Its usefulness should be confirmed by future clinical trials.

#### 2.2.2. *Lactobacillus rhamnosus* can restore the gut microbiota, improve gut barrier function, and decrease levels of pro-inflammatory cytokines

Although *L. rhamnosus* was discovered only in 1983, it has been the most studied and thoroughly researched *Lactobacillus* among all probiotics due to its various roles in the adjuvant treatment of intestinal diseases. *L. rhamnosus* strains CY12, ZFM231, M9, MTCC-5897, LDTM 7511, SHA113, and *L. rhamnosus* GG (LGG) restore the gut microbiota, improve gut barrier function, and improve DSS-induced colitis by downregulating LPS-induced inflammatory cytokines (Son et al., 2019; Fatmawati et al., 2020; Jia et al., 2020; Yeo et al., 2020; Kaur et al., 2021; Pang et al., 2021; Xu et al., 2021; Wan et al., 2022; Zheng et al., 2022). It has been demonstrated that the EPS produced by *L. rhamnosus* plays a significant role in biofilm formation, and as a result, it may successfully repair the injured intestinal mucosa (Capurso, 2019). *L. rhamnosus* FBB81 improves hydrogen peroxide-induced inflammation by enhancing the intestinal epithelial barrier in Caco-2 cells (Fatmawati et al., 2020). A study showed that *L. rhamnosus* HM0539 inhibited the distal NF-κB signaling pathway via TLR4 to attenuate the LPS-induced inflammatory response, with a concentration of 10^8^ CFU/mL having the most significant effect (Li et al., 2020). Additionally, combinations of *L. rhamnosus* with other agents have also been studied. Samat Kozhakhmetov et al. (Kozhakhmetov et al., 2022) mixed *L. rhamnosus* with food-grade horse milk and administered the combination to mice with DSS-induced colitis, resulting in a significant improvement in UC symptoms. Similarly, *L. rhamnosus* 1.0320 with inulin and LGG combined with targose significantly improved symptoms of colitis in mice (Son et al., 2019; Liu et al., 2020). Inulin and tagatose are prebiotics that promote the growth of probiotic bacteria in the intestine. The results suggest that the adherence and dose of probiotics influence the digestive system. Given that the disturbances of the gut microbiota in UC are manifested by the alteration of the quantity and structure of multiple bacteria, simultaneous administration of multiple probiotics may be more effective in restoring the balance of the gut microbiota than administration of a single strain. In clinical trials, 20 patients with UC were given oral LGG for 1 week, resulting in increased concentrations of LGG in the colon, demonstrating the adhesion properties of LGG (Pagnini et al., 2018). However, the observation period in that study was only 1 week; the gut microbiota may not have changed significantly during that period. Additionally, the observed increase in the number of bacteria may have been caused by the oral administration of LGG. Another clinical trial found that 4 weeks of oral administration of a probiotic complex (based on *L. rhamnosus* NCIMB 30174) resulted in a significant decrease in fecal calprotectin levels and a significant improvement in clinical symptoms in patients with UC (Bjarnason et al., 2019). A previous randomized controlled trial demonstrated the long-term effects and safety of LGG in patients with UC (ZOCCO et al., 2006). These findings demonstrate that various *L. rhamnosus* strains, all of which improve UC, have been verified in human *in vivo* investigations and are now recommended probiotics in clinical practice. In the last 5 years, *L. rhamnosus* has been an effective treatment for UC in animal experiments; however, clinical trials in humans are relatively lacking and outdated. Therefore, future research needs to explore other strains by using data of strains with excellent laboratory efficacy in clinical trials.

#### 2.2.3. *Lactobacillus acidophilus* has potential for treating UC due to its anti-inflammatory and gut barrier-protective properties

*Lactobacillus acidophilus* is a gram-positive bacterium that not only produces significant amounts of lactic acid to limit the growth of other dangerous bacteria but is also bile-tolerant, making it a candidate strain for probiotics (Usman and Hosono, 1999). Animal studies have provided the foundation for the treatment of UC by *L. acidophilus*, including ATCC 4356, BIO5768, KBL402, KBL409, and XY27 (Hu et al., 2020; Kim et al., 2021; Hrdý et al., 2022; Li et al., 2022), which all play an effective role in improving gut microbiota, maintaining the intestinal barrier, and lowering levels of inflammatory factors. *L. acidophilus* evidently increased levels of SCFAs, inhibited the NLRP3 inflammasome, and facilitated autophagy to improve UC. Notably, live *L. acidophilus* produces better effects on UC rats than heat-inactivated *L. acidophilus* (Li et al., 2022). However, the improvement in UC symptoms is strain-specific. *L. acidophilus* strains NCFM and FAHWH11L56 improved DSS-induced colitis and increased levels of IL-10 and IL-17 in the colon by altering the CCL2/CCR2 and CCL3/CCR1 axes. The potential alleviating effect of *L. acidophilus* NCFM, CCFM137, and FAHWH11L56 on colitis may be related to the fact that they have an intact gene cluster for the synthesis of EPS (Huang et al., 2022). However, *L. acidophilus* CCFM137 had no therapeutic effects. In contrast, *L. acidophilus* FGSYC48L79 aggravates colitis by increasing the population of harmful bacteria in the gut (Li et al., 2022). Additionally, *L. acidophilus* can extend the lifespan of mice treated with DSS and reduce the severity of colitis by activating M2 macrophages in peritoneal cavity cells and Th2 and Treg cells in splenocytes. Notably, *L. acidophilus* improves UC by modulating the gut microbiota composition and amino acid and oligosaccharide metabolic pathways. The combination with 5-ASA did not affect the pharmacokinetics of *L. acidophilus*, providing strong evidence for its safety (Li et al., 2022). Overall, specific strains of *L. acidophilus* may potentially be used to improve UC. Furthermore, different strains of *L. acidophilus* have varying degrees of effectiveness, which may guide the production of probiotic formulations. The focus of future research should shift from animal studies to human trials to provide new therapy choices for juvenile UC.

#### 2.2.4. *Lactobacillus reuteri* is effective in treating pediatric UC

*Lactobacillus reuteri* is a probiotic found in all vertebrate and mammalian intestines and is one of the few microbial species known to live in the human stomach. *L. reuteri* strongly adheres to the intestinal mucosa and is the only strain that can efficiently eliminate *Helicobacter pylori* (Saviano et al., 2021; Liang et al., 2022). *L. reuteri* also plays a key role in the treatment of colitis. *L. reuteri* ATCC PTA 4659 improved colitis clinically and morphologically in mice (Liu et al., 2022). *L. reuteri* NK33 and NK99 not only significantly attenuated symptoms of colitis but also prevented the occurrence and development of anxiety and depression in mice (Jang et al., 2019). Additionally, pretreatment with *L. reuteri* strains 4,659 (human origin) and R2LC (murine origin) thickened the intestinal mucus and improved DSS-induced colitis in mice (Ahl et al., 2016). Meanwhile, *L. reuteri* R28 showed better colonization than *L. plantarum* AR17-1 (Ahl et al., 2016). *L. reuteri* DSM 17938 increased levels of tryptophan metabolites and purine nucleoside adenosine in neonatal mice as well as increased their tolerance to inflammatory stimuli (Liu et al., 2019). However, the mice in that experiment did not have colitis. Hence, it is unclear whether a reduction in inflammation can prevent the development of UC. *L. reuteri* 1 enhances intestinal epithelial barrier function and lowers the inflammatory response induced by *Enterotoxigenic Escherichia coli* K88 via suppressing the myosin light-chain kinase signaling pathway in IPEC-J2 cells (Gao et al., 2022). Moreover, *L. reuteri* FN041 improves dyslipidemia and repairs mucosal-barrier damage caused by a high-fat diet, and it can also affect the diurnal variation of the gut microbiota (Li et al., 2019). Notably, perinatal mice supplemented with a combination of *L. reuteri* and *L. johnsonii* reduce the incidence of colitis in the neonatal period (Krishna et al., 2022), implying the preventive effect of *L. reuteri* on UC and providing a foundation for the prevention of UC through oral probiotics in children with risk factors. Previous studies have confirmed that *L. reuteri* has multifaceted effects on improving the symptoms of colitis. Further clinical research on *L. reuteri* is needed to explore its use in the treatment of children with UC.

#### 2.2.5. *Lactobacillus gasseri* treats UC by modulating the immune response and gut microbiota

*Lactobacillus gasseri* tolerates low pH and bile salt environments and is strongly adherent, which provides the foundation for its successful colonization of the human intestine. This results in a variety of benefits via antimicrobial activity, bacteriocin production, and immunomodulation by innate and adaptive systems (Selle and Klaenhammer, 2013). *L. gasseri* 4 M13 fermented with protein and galactose regulates the systemic inflammatory response and improves the intestinal epithelial barrier (Jeong et al., 2022). *L. gasseri* G098 modulates host immunity and the gut microbiome to improve colitis symptoms in mice (Zhang et al., 2022). *L. gasseri* NK109 improves gut dysbiosis and alleviates symptoms of both colitis and depression (Yun et al., 2020). *L. gasseri* RW2014 modulates the metabolism of bile acid and the composition of the gut *microbiota* (Li et al., 2022). Furthermore, co-administration of *L. gasseri* KBL697 and infliximab has a synergistic effect on treating colitis in mice by decreasing levels of pro-inflammatory cytokines (Han et al., 2022). *L. gasseri* M1 increases levels of SCFAs to repair intestinal barrier damage caused by DSS in mice (Cheng et al., 2023). Moreover, *L. gasseri* can inhibit the expression of the TNF-α-converting enzyme in host cells to suppress the release of TNF and IL-6 (Gebremariam et al., 2019). However, the precise function of *L. gasseri* is mostly unknown and requires further research. Nevertheless, *L. gasseri* has anti-inflammatory properties that improve gut health. However, there is limited evidence that *L. gasseri* is effective in the treatment of pediatric UC.

#### 2.2.6. *Lactobacillus paracacei* maintains and improves intestinal barrier function

*Lactobacillus paracacei* is a gram-positive parthenogenic anaerobic bacterium with high acid and bile salt tolerance that can enhance immunity. *L. paracacei* NTU101 strengthens antioxidant capabilities to protect mice from DSS-induced colitis (Chen et al., 2019). *L. paracacei* R3 significantly attenuates pathological damage and symptoms of colitis by regulating Th17/Treg cell balance (Huang et al., 2021). Additionally, *L. paracacei*-derived extracellular vesicles also reduce the expression of pro-inflammatory cytokines by augmenting the pathway of endoplasmic reticulum stress (Choi et al., 2020). Meanwhile, *L. paracacei* BD5115 promotes the proliferation of intestinal epithelial cells (Qiao et al., 2022). Notably, neonatal administration of *L. paracacei* N1115 prevents intestinal inflammation in adulthood in mice (Xun et al., 2022). A randomized controlled clinical trial has shown that oral administration of *L. paracacei* CBA L74-fermented formula could enhance the immune system, microbiota, and metabolome maturation in infants (Roggero et al., 2020) and that *L. paracacei* BD5115 may help repair intestinal damage. In summary, *L. paracacei* exhibits protective effects on the intestine, especially in maintaining the stability of the intestinal barrier. However, more studies are needed to clarify whether *L. paracacei* can improve symptoms of UC by improving intestinal barrier function.

#### 2.2.7. *Lactobacillus johnsonii* treats colitis by decreasing the level of pro-inflammatory cytokines

According to the FDA, *L. johnsonii* is a naturally occurring strain in the human digestive tract. *L. johnsonii* prevents colonic shortening and spleen augmentation and attenuates colonic hyperplasia by reducing levels of inflammatory factors in mouse colitis models (Zhang et al., 2021). *L. johnsonii* improved experimental colitis by promoting the conversion of native macrophages into CD206+ macrophages and releasing IL-10 via the TLR1/2-STAT3 pathway (Jia et al., 2022). Moreover, *L. johnsonii* repairs the TJ of Caco-2 cells damaged by hydrogen peroxide and enhances barrier function and integrity (Bai et al., 2022). In a piglet Salmonella model with diarrhea, *L. johnsonii* L531 improved enteritis by removing damaged mitochondria (Xia et al., 2020). Taken together, *L. johnsonii* has significant potential for treating UC due to its anti-inflammatory properties. However, there is scarce evidence related to the treatment of UC in children using *L. johnsonii*; hence, further research is needed to confirm its efficacy.

#### 2.2.8. *Lactobacillus kefiranofaciens* improves intestinal homeostasis in piglets

*Lactobacillus kefiranofens* is a gram-positive parthenogenic anaerobic bacterium that generates EPS derived from Kefir grains. The precise categorization of *L. kefiranofens* is unknown (Chen et al., 2017). However, *L. kefiranofens* ZW18 modulates the gut microbiota (Zhao et al., 2022). Additionally, *L. kefiranofens* JKSP109 alleviates inflammation, prevents colorectal carcinogenesis, and reduces the DAI in mice with colitis (Zhao et al., 2022). Furthermore, *L. kefiranofens* BS15 enhances intestinal immunity and the gut microbiota, resulting in an improved diarrhea index in piglets (Xin et al., 2020). Some studies showed that environmental factors, such as heat, cold, acid, and bile salts, could affect the activity of *L. kefiranofens*; hence, encapsulation of *L. kefiranofens* must be performed to preserve its beneficial effects (Wang et al., 2015; Chen et al., 2017). Although *L. kefiranofens* can potentially improve intestinal homeostasis and is a potential probiotic, future studies need to explore its role in maintaining intestinal homeostasis.

#### 2.2.9. *Lactobacillus helveticus* enriches the gut microbiota and increases levels of anti-inflammatory cytokines

*Lactobacillus helveticus* is a gram-positive bacterium with high proteolytic activity. Its ability to synthesize EPS can reduce the number of harmful bacteria such as *Clostridium perfringens* and increase the number of parabacteria to enhance the ability of symbiotic bacteria in the intestine to produce SFAs (Wang et al., 2022). *L. helveticus* KLDS 1.8701 has antibacterial, antioxidant, and immunomodulatory capacity in DSS-induced colitis mouse models (Shi et al., 2021). *L. helveticus* ASCC 511, an intestinal commensal high in guanine (L-citrulline), dramatically ameliorates DAI, reduces colonic tissue damage, and reduces levels of pro-inflammatory markers in mice (Ho et al., 2022). Additionally, oral intake of *L. helveticus* NS8 significantly inhibits the activation of NF-κB and upregulates IL-10 (Rong et al., 2019). Although current research on *L. helveticus* does not focus on its use for the treatment of UC, its potential as a probiotic should be explored further given its important function in altering the gut microbiota and increasing levels of anti-inflammatory cytokines.

#### 2.2.10. *Lactobacillus fermentum* improves intestinal inflammation and alleviates symptoms of UC

*Lactobacillus fermentum* is a gram-positive bacterium with significant acid-producing capacity. *L. fermentum* also has high acid tolerance and can inhibit pathogens by producing antimicrobial peptides. In humans, *L. fermentum* may potentially improve metabolic and immune diseases because it can significantly improve the gut microbiota (Molina-Tijeras et al., 2021). Several strains may improve DSS-induced colitis in mice. *L. fermentum* MTCC 5689, KBL374, KBL375, and a hybrid strain containing *L. fermentum* L930BB all had positive effects on the remission of colitis without causing any serious side effects in mice (Paveljšek et al., 2018; Pradhan et al., 2019). *L. fermentum* F-B9-1 protects the intestinal barrier and has shown anti-inflammatory potential by alleviating DSS-induced experimental UC in mice, and the EPS from the bacterium act as active components to inhibit inflammation by reducing the levels of IL-1β and IL-6 (Pradhan et al., 2019). *L. fermentum* HFY-06 ameliorates the pathological damage induced by DSS by balancing the ratio of anti- and pro-inflammatory cytokines (Liu et al., 2022). Similarly, *L. fermentum* ZS-40 has anti-inflammatory effects (Chen et al., 2021). It is probable that after establishing efficacy in human clinical trials, it can be safely used as a biological therapy to improve inflammation in UC.

#### 2.2.11. *Lactobacillus coryniformis* has antioxidant properties

*Lactobacillus coryniformis* is a gram-positive bacterium that is primarily found in the respiratory and reproductive systems of humans. *L. coryniformis* can be used in human food (Lara-Villoslada et al., 2007); surprisingly, it is frequently utilized as a vaccine adjuvant. *L. coryniformis* MXJ32 improves intestinal barrier function, enhances beneficial gut microbiota, and reduces levels of pro-inflammatory factors; however, it has not yet been tested on colitis models (Wang et al., 2022). Meanwhile, *L. coryniformis* NA-3-derived EPS has the ability to scavenge free radicals and is therefore expected to be used as an antioxidant in the treatment of patients with ulcerative colitis (Xu et al., 2020). The specific function of *L. coryniformis* remains unknown and requires additional exploration. Although *L. coryniformis* has anti-inflammatory effects and improves gut health, evidence that *L. coryniformis* is effective in the treatment of UC disease is limited.

#### 2.2.12. *Lactobacillus curvatus* has anti-inflammatory effects

*Lactobacillus curvatus* is a gram-positive bacterium found primarily in the urinary tract and has high antioxidant capability. EPS-producing *L. curvatus* strains can prevent the formation of *Salmonella enterica* serovar Eteritidis biofilm and effectively limit the colonization of pathogenic bacteria (Redondo et al., 2017). In animal investigations, blood levels of IL-6, TNF-R1, TNF-R2, and TNF-α were considerably reduced in mice administered with *L. curvatus* BYB3, which improved DSS-induced intestinal inflammation (Wang et al., 2022). Meanwhile, *L. curvatus* GH5L demonstrates antioxidant effects (Düz et al., 2020). Current studies suggest that *L. curvatus* has anti-inflammatory and antioxidant properties, but there is no evidence that it can be used to improve symptoms of UC. Because *L. curvatus* is not abundant in the intestine, more animal and clinical trials are needed to determine how effective oral *L. curvatus* is in treating UC and whether it has harmful effects.

#### 2.2.13. *Lactobacillus delbrueckii* improves the intestinal barrier and regulates immunity

*Lactobacillus delbrueckii* is a gram-positive bacterium that has received little attention as an adjuct for the treatment of diseases. However, *L. elbrueckii* reduces LPS-induced damage of the intestinal epithelium in piglets and improves mucosal barrier function (Düz et al., 2020). Furthermore, in mice with colitis, *L. elbrueckii* improved symptoms aggravated by alcohol (Cannon et al., 2022). However, whether this is attributed to the ability of *L. elbrueckii* to counteract the effects of alcohol or the influence of other actions is uncertain. Oral administration of *L. elbrueckii* can improve intestinal integrity by strengthening the intestinal structure and TJ while increasing antioxidant activity via the TLR-nuclear factor erythroid 2-related factor 2 signaling pathway in piglets (Cannon et al., 2022). *L. elbrueckii* also improves gut immunity in suckling piglets by activating dendritic cells (Peng et al., 2022). Additionally, *L. elbrueckii* modulates immunity through different mechanisms while also having anti-inflammatory effects. Unfortunately, the therapeutic effect of *L. elbrueckii* on UC remains unclear. *L. elbrueckii* may be a natural probiotic that inhibits the development of UC.

### 2.3. Effects of other probiotics on UC

*Bacillus* spp. is found in all living species and the environment, and it has considerable applications in agriculture, industry, medicine, and health. *Bacillus spp.* is resistant to external damage and suited for gastrointestinal digestion, storage, and survival. *Bacillus amyloliquefaciens* improves symptoms of TNBS-induced colitis in mice by attenuating the expression of pro-inflammatory cytokines (Khalifa et al., 2022). *Bacillus cereus* enhances intestinal barrier function and modulates the gut microbiota to improve symptoms in mice with colitis (Sheng et al., 2021). *Bacillus smithii* XY1 has anti-inflammatory properties that can attenuate the inflammatory response (Huang et al., 2021). *Bacillus subtilis* improves colitis in experimental UC mice by maintaining the integrity of the intestinal barrier and suppressing inflammatory responses (Chung et al., 2021; Zhang et al., 2021). Previous studies have demonstrated the therapeutic effects of *Bacillus spp.* in mice with colitis. *Bacillus* is a potential candidate probiotic for the treatment of UC, but more clinical trials are needed to support its use for UC.

*Pediococcus* is mainly found in fermented plant materials and pickled vegetables. *Pediococcus pentosaceus* showed protective effects on the intestinal tract. *Pediococcus pentosaceus* belongs to the genus *Pediococcus*, which can ferment glucose to produce lactic acid. *Pediococcus pentosaceus* CECT 8330 regulates immunity and the gut microbiota to improve DSS-induced colitis in mice (Dong et al., 2022). *Pediococcus pentosaceus* LI05 modulates immunological profiles, the gut microbiota, and SCFA levels in mice (Bian et al., 2020). Oral administration of *Pediococcus pentosaceus* SMM914 can activate the Nrf2-Keap1 antioxidant signaling pathway to increase antioxidant capacity in piglets (Bian et al., 2020). *Pediococcus pentosaceus* improves symptoms of colitis in animal experiments and could be a candidate probiotic for the treatment of pediatric UC. However, the specific functions of other strains of *Pediococcus* in UC remain unclear and require further investigation.

*Escherichia coli* Nissle1917 (EcN) belongs to the non-pathogenic gram-negative bacteria of the Enterobacteriaceae family (Schultz, 2008). Currently, EcN has been used for the treatment of UC with considerable efficacy. A randomized controlled trial verified that 5-ASA combined with EcN can improve the quality of life of patients with UC and induce colonoscopic remission (Park et al., 2022). EcN can repair and maintain the integrity of the intestinal epithelium, regulate the host immune response, and modulate the gut microbiota (Schlee et al., 2007). The role of EcN in the treatment of UC is well established, and future research should focus on the use of bioengineering to improve the stability of gene expression and enhance the therapeutic efficacy of EcN.

### 2.4. Effects of multi-strain probiotics on UC

Apart from a single strain, we also discuss the effectiveness of simultaneous combinations of probiotics, including two or more strains. The mixed preparation of *B. longum* Bif10 and *B. breve* Bif11 increased anti-inflammatory marker levels and SCFAs, ameliorating symptoms of colitis in mice (Sharma et al., 2023). In addition, a mixture of *L. rhamnosus* NCIMB 30174, *L. plantarum* NCIMB 30173, *L. acidophilus* NCIMB 30175, and *Enterococcus faecium* NCIMB 30176 decreased intestinal inflammation and reduced fecal calprotectin in adults with UC (Bjarnason et al., 2019). A mixture of *L. johnsonii* IDCC9203, *L. plantarum* IDCC3501, and *B. lactis* IDCC4301 reduced intestinal histological damage, such as loss of goblet cells, immune cell infiltration in the mucosa, and submucosal and crypt destruction (Je et al., 2018). Moreover, mixed lactobacilli had superior anti-inflammatory effects compared with single-strain treatments (Shi et al., 2021). The probiotic product Bifico, composed of *B. longum*, *L. acidophilus*, and *Enterococcus faecalis*, increased the expression of TJ proteins and the number of Tregs (Zhang et al., 2018). Additionally, oral *Bifico* enhanced the efficacy of mesalazine and maintained the UC in remission (Fan et al., 2019). Other related mixed microbial preparations have also achieved positive effects in animal tests (Li et al., 2022). Furthermore, mixed probiotics can significantly improve the quality of life of patients (Kamarli Altun et al., 2022). Therefore, complex probiotics may potentially be used to relieve symptoms of UC. However, the effectiveness of combined probiotics should be interpreted cautiously. In an animal experiment, consumption of Bifico might exacerbate the damage of colonic tissue when the mucosal barrier is impaired (Zhang et al., 2018). Therefore, we believe that blended probiotics are not appropriate for individuals with UC with severe symptoms and intestinal mucosal tissue destruction. Moreover, the effects of probiotics depend on both strains and dose; therefore, the proportion of each strain in mixed probiotics and the dose of probiotics can influence their effects, which is a problem that needs to be solved in the future.

The above extensive animal studies have explored the role of potential probiotics in the treatment of UC, providing us with additional therapeutic options. However, to clarify whether these probiotics can treat pediatric UC, the findings from animal studies need to be applied to clinical trials, which is the direction of our future exploration. Due to the poor controllability and low follow-up rate of pediatric UC patients, clinical trials on probiotics for pediatric UC have been scarce. As shown in Table 3, we found only three relevant clinical studies about children with UC and added the clinical studies in adults with UC, hoping to provide useful reference data for the role of probiotics in the treatment of pediatric UC. Based on previous clinical studies in adults, the probiotic most commonly used in clinical studies is currently VSL#3 (a probiotic including eight strains: *Lactobacillus casei*, *L. plantarum*, *L. acidophilus*, *L. delbrueckii* subsp. *bulgaricus*, *Bifidobacterium longum*, *B. breve*, *B. infantis*, and *Streptococcus salivarius* subsp). Supplementation of conventional therapy with oral VSL#3 can induce and maintain remission in active UC by modulating the gut microbiota structure and improving the intestinal mucosal barrier. The increased remission rate and significantly lower relapse rate at follow-up within the period of VSL#3 administration in both adult and child patients with UC demonstrate the effective therapeutic effect of probiotics. In addition, the combination of *Bifidobacterium* trivium capsules with mesalazine significantly reduced DAI and clinical symptom scores in patients with UC while decreasing the level of inflammatory factors and increasing the level of IL-10, which has an inhibitory effect on inflammation (Agraib et al., 2022). In a study in which patients with UC received probiotic therapy for 2 years, it was noted that long-term administration of probiotics could replace glucocorticoids in the treatment of mild to moderate UC (Palumbo et al., 2016). In addition to oral administration, probiotic rectal administration using enemas can also achieve relief of UC symptoms (Matthes et al., 2010; D’Incà et al., 2011). However, it is not clear which mode of administration provides the best effect. Some patients with UC undergo total proctocolectomy with ileal pouch anal anastomosis (IPAA); pouchitis is a common postoperative complication, and there are many studies showing the beneficial role of probiotics in the prevention and treatment of pouchitis (Veereman-Wauters, 2003; Gionchetti et al., 2007; Pronio et al., 2008). The application of probiotics (La-5) and *Bifidobacterium* (Bb-12) after IPAA can increase the number of intestinal *Lactobacillus* and *Bifidobacterium* in patients, reduce involuntary bowel movements, abdominal cramps, and endoscopic scores (Laake et al., 2005), and it has also been shown that VSL#3 can improve joint pain in UC patients with extraintestinal manifestations (Karimi, O., et al., 2005). During all trials, no probiotic-related side effects were reported in patients, demonstrating the good safety profile of probiotics. It is evident from these clinical studies that probiotics have good adjuvant therapeutic effects and are more effective in inducing mild to moderate UC. The positive results of adult studies are inspiring and informative for pediatric studies and bring hope to pediatric patients with UC. However, children are not the epitome of adults, and caution should be exercised when drawing on adult-related research results. Additionally, many of the current trials are pilot studies; to further investigate the role of probiotics in the treatment of UC in children, more large samples and high-quality randomized controlled trials deserve to be investigated.

**Table 3:** Effects of Probiotics on the clinical manifestations of UC.

Probiotics	Participants	Intervention	Results	Reference
VSL#3	14 consecutive patients with UC aged between 1.7 and 16.1 years	450–1,800 × 10^9^ bacteria daily for 1 year	Effective in maintaining of remission.	Miele et al. (2009)
VSL#3	9 patients with mild to moderate acute UC aged 3–17 years	450 × 10^9^ bacteria twice daily for 8 weeks	Effective in inducting of remission.	Huynh et al. (2009)
*Lactobacillus reuteri* ATCC 55730	40 patients with mild to moderate UC aged 6–18 years	10× 10^9^ CFU for 8 weeks	Improving mucosal inflammation	Oliva et al. (2012)
*Bifidobacterium* triplex live bacteria	80 UC patients aged 22 to 65 years	oral mesalazine (1.5 g/d) combined with bifid triple viable (1.26 g/d) for 2 months.	The overall response rate of treatment group (91.11%) was significantly higher than that of control group (68.89%)	Jiang et al. (2020)
*Bifidobacterium breve* strain Yakult (BFM)	98 patients with UC aged 20–70 years	one pack of *B. breve* strain Yakult fermented milk per day for 48 weeks	BFM had no effect on time to relapse in UC patients compared with placebo.	Matsuoka et al. (2018)
*Lactobacillus rhamnosus* GG	20 patients with UC Unknown age	“double” dose of LGG for a week	Reduced expression of TNFα and IL-17, increased mucosal concentrations.	Pagnini et al. (2018)
Combined probiotics: *Lactobacillus rhamnosus* NCIMB 30174, *Lactobacillus plantarum* NCIMB30173, *Lactobacillus acidophilus* NCIMB30175 and *Enterococcus faecium* NCIMB 30176	81 patients with UC aged 18–70 years	1 mL/kg/day for 4 weeks	Decreased intestinal inflammation in patients with UC	Bjarnason et al. (2019)
*Escherichia coli Nissle* 1917	58 patients with UC aged 46.3 ± 14.1 years	2.5 × 10^9^ CFU/ capsule, one capsule/day from day 1 to day 4 and two capsules/ day from day 5 for 8 weeks.	Increased inflam matory bowel disease questionnaire (IBDQ), higher number of patients displayed clinical response at 4 weeks, endoscopic remission at 8 weeks	Arena et al. (2017)
a symbiotic composed of *Lactobacillus acidophilus, Lactobacillus* *plantarum, Enterococcus faecium, Bifidobacterium longum, Bifidobacterium lactis*, and *Streptococcus thermophiles*	20 patients with UC aged 44.94 ± 14.14 years	one tablet a day for 8 weeks	Increased QoL scores	Liu et al. (2021)
Capsule containing nine *Lactobacillus* and five *Bifidobacterium* species	24 patients with mild-tomoderate UC aged between 18 and 65 years	3 capsules daily for 6 weeks	Induced remission in UC patients.	Agraib et al. (2022)
*Bifidobacterium longum* 536	56 patients with mild-tomoderate UC aged 44.9 ± 14.5 years	200–300 × 10^9^ freeze-dried viable three daily for 8 weeks	Reducing UCDAI scores, EI scores and the Mayo subscore.	Tamaki et al. (2016)
Capsule (containing *Lactobaccillus acidophilus* LA-5, *Lactobacillus delbrueckii subsp. bulgaricus* LBY-27, *Bifidobacterium animalis subsp. lactis* BB-12 and *Streptococcus thermophilus* STY-31)	8 patients with mild-tomoderate UC aged between 50 and 65 years	3 capsules daily for 1 month	No significant differences	Ahmed et al. (2013)
VSL#3	22 patients with mild-tomoderate UC aged between 20 and 70 years	4 sachets daily for 8 weeks	Effective in achieving clinical responses and remissions in patients with mild-to moderately active UC	Lee et al. (2012)
Capsule (containing *Lactobacillus acidophilus* La-5 and *Bifidobacterium animalis subsp. lactis BB-12*)	20 patients with mild-tomoderate UC in remission aged ≥18 years	two capsules three times daily for 52 weeks	No significant differences	Wildt et al. (2011)
*Bifidobacterium*	21 patients with mild-tomoderate UC aged 43.6 ± 13.2 years	probiotic powder (10^9^ CFU/g) three times a day for one year	Improved the clinical condition of patients with UC.	Ishikawa et al. (2011)
*Lactobacillus casei DG*	18 patients with mild-tomoderate UC (unknown age)	rectally administered 8–9 10^8^ cfu twice daily for 8 weeks	Modified colonic microbiota	D’Incà et al. (2011)
VSL#3	71 patients with mild-tomoderate UC aged >18 years	3,600 × 10^9^ CFU daily for 8 weeks	Reduced UCDAI scores	Tursi et al. (2010)
VSL#3	77 patients with mild-tomoderate UC aged 39.8 ± 13 years	twice daily for 12 weeks	Effective in achieving clinical responses and remissions	Sood et al. (2009)
VSL#3	20 and 34 patiens with UC, respectively, (unknown age)	for 6 weeks and for 12 months (unknown dose)	Effectively induced and maintained remission	Chapman et al. (2007)
VSL#3	32 ambulatory patients with active UC aged 35 ± 13 years	3,600 × 10^9^ bacteria daily for 6 weeks	Effective in inducting of remission.	Bibiloni et al. (2005)
VSL#3	20 patients with UC aged 22–64 years	6 g once daily for one year	Effective in maintaining of remission.	Mimura et al. (2004)
*Lactobacillus delbruekii* and *Lactobacillus fermentum*	15 patients with mild-tomoderate UC aged 48 ± 1.90 years	10 × 10^9^ CFU daily For 8 weeks	Helpful in maintaining remission and preventing relapse of UC.	Hegazy and El-Bedewy (2010)
*Lactobacillus* GG	65 patients with quiescent UC aged 34 ± 6 years	18 × 10^9^ viable bacteria/day for 12 months	Effective and safe for maintaining remission	ZOCCO et al. (2006)
BGS (produced by *Propionibacterium freudenreichii*)	12 patients with UC aged 18–49 years	orally 4.5 g of BGS daily for 4 weeks	Clinical and endoscopic improvements in patients with UC	Suzuki et al. (2006)
*Bifidobacterium longum*	8 patients with UC aged 24–67 years	200 × 10^9^ twice daily for 4 weeks.	Improved the clinical appearance	Furrie et al. (2005)
*Escherichia coli Nissle* 1917	162 patients with UC aged 19–69 years	200 mg once daily for 12 months	Effective in maintaining of remission	Kruis et al. (2004)
BIFICO	15 patients with UC (unknown age)	1.26 g daily for 8 weeks	effective in maintaining remission and preventing relapse	Cui et al. (2004)
Kefir	15 patients with UC aged 19–68 years	400 mL daily for 4 weeks	May modulate gut microbiota, may improve the patient’s quality of life in the short term	Yılmaz et al. (2019)
Profermin (including *Lactobacillus plantarum* 299v)	39 patients with mild to moderate UC aged 18–50 years	twice daily for 24 weeks	Safe and may be effective in inducing remission of active UC	Krag et al. (2012)
*Bifidobactrium longum* B536	14 patients with UC aged 43 ± 5 years	200–300 × 10^9^ daily for 24 weeks	Achieved clinical remission	Takeda et al. (2009)
BFM	10 patients with mild to moderate, active UC mean aged 30.2 years	100 mL daily of BFM for 12 weeks	Safe and more effective than conventional treatment alone	Kato et al. (2004)

## 3. Potential mechanisms of probiotics in the treatment of UC

**Figure 3:**
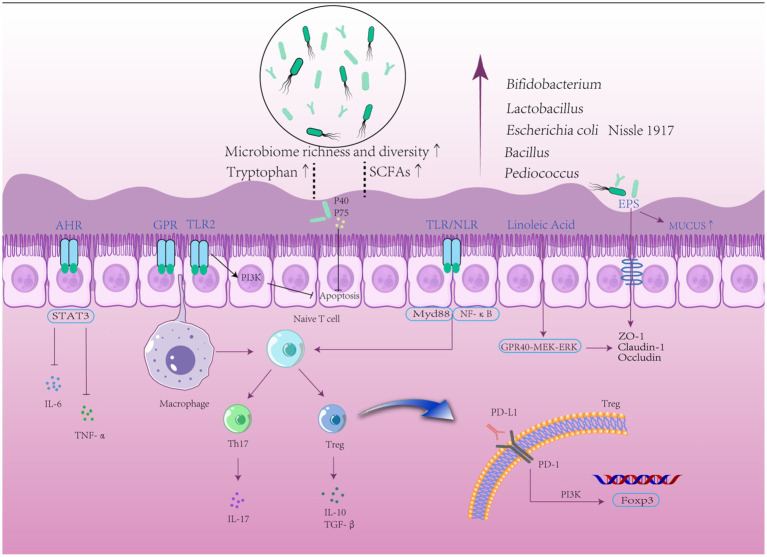
Protective mechanisms of probiotics. Probiotics can maintain gut microbiota homeostasis by increasing the number of beneficial bacteria, regulating T cell differentiation to control inflammatory responses and immune disorders, and maintaining barrier function by regulating signaling pathways to upregulate the expression of tight junction proteins.

As shown in Figure 3, the mechanism of probiotics for UC treatment is three-fold: regulating gut microbiota homeostasis, improving intestinal barrier function, and modulating the intestinal immune response. The three are interrelated and interact with each other to promote the remission of UC in terms of pathology and symptoms. Additionally, probiotics’ unique characteristics depend on the strain level; therefore, we list detailed information on the mechanism of action of different strains in Tables 4, 5.

**Table 4:** Mechanisms of action of *Bifidobacterium* in the treatment of UC.

Probiotics	Strain	Mechanisms of action	Reference
*Bifidobacterium longum*	YS108R	Down-regulated IL-6 and IL-17A both at serum and mRNA levels and maintained the tight junction proteins	Yan et al. (2019) and Yan et al. (2020)
	5^1A^	Reduced intestinal permeability and lowered IL-1β, myeloperoxidase, and eosinophil peroxidase levels	Abrantes et al. (2020)
	LC67	Down-regulated Th17 cell differentiation and IL-17 expressions, and upregulated Treg cell differentiation and FoxP3 and IL-10 expressions	Jang et al. (2018) and In Kim et al. (2019)
	HB5502	Inhibition of high mobility group box 1(HMGB1) release and subsequent HMGB1-mediated gut barrier dysfunction	Chen et al. (2019)
	DD98	Inhibited the activation of the TLR4 and increased the expression of tight junction proteins including ZO-1 and occluding	Hu et al. (2022)
	NK151, NK173, and NK175	Decreased blood LPS, IL-6, and creatinine levels	Yoo et al. (2022)
	CECT 7894	Regulated the gut microbiota composition and bile acid metabolism	Xiao et al. (2022)
*Bifidobacterium breve*	H4-2 and H9-3	Inhibited the expression of the NF-κB signaling pathway, increased the levels of SCFAs and improved gut microbiota	Niu et al. (2022)
	CCFM683 and BJCP1M6	Decreased the pro-inflammatory cytokines, maintained the concentration of intestinal TJ proteins and AJ proteins, decreased epithelial cell apoptosis	Chen et al. (2021)
*Bifidobacterium lactis*	A6	Decreasing MDA and increased SOD and GSH levels in colon tissues, down-regulated TNF-α, IL-1β and IL-6 levels and upregulated IL-10 level in colon tissues	Wang et al. (2022)
	BB12	Modulated pro-apoptotic cytokine expression	Chae et al. (2018)
*Bifidobacterium bifidum*	FJSWX19M5	Promoted Treg cells differentiation and intestinal barrier restoration and induced higher IL-10 levels and a lower ratio of IL-22/IL-10 and IL-17/IL-10	Qu et al. (2023)
	FL-276.1 and FL-228.1	Activated the aryl hydrocarbon receptor (AhR) in the intestine to down-regulate TNF-α, IL-1β and IL-6	Cui et al. (2022)
	WBIN03	Down-regulated the pro-inflammatory cytokines and upregulated antioxidant factors	Wang et al. (2018)
*Bifidobacterium infantis*	ATCC 15697	Reduced the NLRP3 inflammasome mRNA level and increased ZO-1, occludin, and claudin-1 tight junction molecule mRNA levels	Sheng et al. (2020)
	FJSYZ1M3	Decreased the levels of inflammatory cytokines IL-6, IL-1β, and IL-10, ameliorated gut microbiota disturbance and increased the level of butyric acid in cecal contents	Li et al. (2023)
*Bifidobacterium adolescentis*	NK98	Suppressed NF-κB action, reduced the Proteobacteria population and increased the Clostridia population	Jang et al. (2019) and Han et al. (2020)
	Reuter 1963	Inhibited of TLR4/NF-B signaling and inducted of intestinal Pglyrp3 production	Ghadimi et al. (2021)
	ATCC15703	Stimulated protective Treg/Th2 response and gut microbiota remodeling	Fan et al. (2021)
	IF1-11 and IF1-03	Suppressed simmune responses in the gut via the macrophage-regulated Treg/Th17 axis.	Yu et al. (2019)

**Table 5:** Mechanisms of action of *Lactobacillus* in the treatment of UC.

Probiotics	Strain	Mechanisms of action	Reference
*Lactobacillus plantarum*	AR17-1	Inhibited the upregulation of IL-1β, TNF-α and IL-6	Wang et al. (2021)
	Q7	Ameliorated the immune response by regulating the TLR4-MyD88-NF-kB pathway	Hao et al. (2021)
	Y44	Enhanced expressions of colonic tight junction proteins and manipulated the ratio of Firmicutes/Bacteroidetes (F/B) and Proteobacteria relative abundance at the phylum level	Gao et al. (2021)
	12	Improved immunity via activating the JAK–STAT pathway and up-regulating adenosine deaminase and interferon-induced protein with tetratricopeptide repeats 1 protein, and upregulated mucin 2 (MUC2) protein expression	Sun et al. (2020)
	N13 and CCFM8610	Reduced expression of pro-inflammatory cytokines, and significantly inhibited expression of p65	Liu et al. (2020)
	CAU1055	Reduced levels of inducible nitric oxide synthase, cyclooxygenase 2, TNF-α, and IL-6.	Choi et al. (2019)
	L15	Increased ZO-1, Occludin, and Claudin-1, and MUC2 mRNA expression levels, improved gut microbiota composition and increased SCFAs, and reduced the transfer of NFκB p65 to the nucleus	Wang et al. (2022)
	HNU082	Reduced intercellular cell adhesion molecule-1, vascular cell adhesion molecule, increased goblet cells and mucin2, increased ZO-1, ZO-2 and occludin, decreased claudin-1 and claudin-2, reduced the content of IL-1β, IL-6, TNF-α, MPO, and IFN-g and increased IL-10, TGF-β1, and TGF-β2 and inhibited the NF-κB signaling pathway	Wu et al. (2022a) and Wu et al. (2022b)
	ZS62	Downregulate the levels of MDA, MPO, IL-1β, IL-6, IL-12, TNF-α, and IFN-γ and the relative mRNA and protein expression of IL-1β, IL-12, TNF-α, COX-2, iNOS, and NF-κB p65, and upregulate the serum levels of CAT, T-SOD, and IL-10 and the relative mRNA and	Pan et al. (2021)
	06 cc2	Induced il-10 production in the colon and attenuated colon inflammation	Tanaka et al. (2020)
	CBT LP3	Increased induction of regulatory T cells and Th2 cells in splenocytes and restored the goblet cells	Kim et al. (2020)
	MTCC 5690	Improved intestinal permeability and decreased MPO activity	Pradhan et al. (2019)
	YS3	Upregulated the expression levels of c- Kit and stem cell factor, downregulated CXCR2 and IL- 8 and increased in the GSH content and IL- 2	Hu et al. (2021)
	CQPC06	Increased the mRNA and protein expression levels of endothelial nitric oxide synthase, neuronal nitric oxide synthase, and nuclear factor of kappa light polypeptide gene enhancer in B-cells inhibitor alpha, decreased the expression levels of inducible nitric oxide synthase, NF-κB, CXCR1, and CXCR2	Zhang et al. (2018)
	QS6-1, QHLJZD20L2 and VJLHD16L1	The expression of ZO-1 and occludin mRNA in the colon markedly decreased	Liu et al. (2022)
	AR326	Restored of the tight junction protein expression and reducted of the abnormal expression of pro-inflammatory cytokines	Wang et al. (2019)
	CCFM242	Enhanced levels of mRNA expression of colonic TJ, increased modulatory effects on the anti-oxidant and immune defense systems in levels of SCFAs	Zhai et al. (2019)
	AB-1 and SS-128	Maintained intestinal health by regulating intestinal flora	Qian et al. (2022)
	IMAU10216, IMAU70095 and IMAU 10,120	Decreased the level of pro-inflammatory cytokines and increased the level of anti-inflammatory cytokines	Khan et al. (2022)
	ZDY2013	Down-regulated the pro-inflammatory cytokines and upregulated antioxidant factors	Wang et al. (2018)
	LC27	Down-regulated Th17 cell differentiation and IL-17 expressions, and upregulated Treg cell differentiation and FoxP3 and IL-10 expressions	Jang et al. (2018) and In Kim et al. (2019)
*Lactobacillus rhamnosu*	MTCC-5897	Diminished levels of pro-inflammatory markers and enhanced levels of the anti-inflammatory cytokine TGF-β with IgA	Kaur et al. (2021)
	LDTM 7511	Increased the expression of claudin-2	Yeo et al. (2020)
	SHA113	Increased of total SCFA production, down-regulated of the expression of TNF-α, IL-6, and IL-1β, upregulated of the expression of the IL-10 and upregulated of the expression of mucins and ZO-1	Pang et al. (2021)
	GG	Down-regulated the TLR4-MyD88 axis as well as of the downstream MyD88dependent activated NF-κB signaling, decreased the IL-17+ Th17 proportion and increased the CD25+ Foxp3+ Treg proportion via the TLR2 pathway, reduced expression of TNFα and IL-17	Son et al. (2019), Jia et al. (2020), Li et al. (2020), and Pagnini et al. (2018)
	1.0320	Regulate the expression levels of inflammatory cytokines IL-1β, IL-6, TNF-α and IL-10, increase the abundance and diversity of gut microbiota	Liu et al. (2020)
	KLDS 1.0386	Upregulated AHR mRNA expression to activate the IL-22/STAT3 signaling pathway	Shi et al. (2020)
*Lactobacillus acidophilus*	ATCC4356	Increased the contents of SCFAs, inhibited NLRP3 inflammasome and facilitated autophagy	Li et al. (2022)
	BIO5768	Enhanced their ability to support the secretion of IL-17 by CD4+ T cells and increased the production of IL-22 by type 3 innate lymphoid cells	Hrdý et al. (2022)
	KBL402 and KBL409	Altered expressions of inflammation-related micro-RNAs (miRs) and restored the diversity of the gut microbiota	(Kim et al., 2021)
	XY27	Increased the gene expression levels of ZO-1, NF-κB, p53, and IκB-α	Hu et al. (2020)
	FAHWH11L56, FGSYC48L79,	Increasing the IL-10 and IL-17 in the colon, and modifying the CCL2/CCR2 axis and CCL3/CCR1 axis	Huang et al. (2022)
*Lactobacillus reuteri*	R28	Inhibited the upregulation of IL-1β, TNF-α and IL-6	Wang et al. (2021)
	R2LC and ATCC PTA 4659	Increased the tight junction proteins occludin and ZO-1 in the bottom of the colonic crypts	Ahl et al. (2016)
	ATCC PTA 4659	Prevented the CD11b + Ly6G+ neutrophil recruitment, dendritic cell (DC) expansion and Foxp3 + CD4+ T-cell reduction, increased TJ proteins and cytoprotective heat shock protein (HSP) 70 and HSP25	Liu et al. (2022)
	DSM 17938	Inhibiting the Toll-like receptor 4-mediated NF-κB pathway, facilitated the induction of immune-modulating Tregs, and lowered proinflammatory effector-memory T cells	Liu et al. (2019)
	R2LC	Increased the PD-1-T follicular helper cell-dependent IgA induction and production	Liu et al. (2021)
*Lactobacillus gasseri*	G098	Improved serum cytokine balance and increased gut microbiota diversity	Zhang et al. (2022)
*Lactobacillus paracasei*	NTU 101	Improved anti-oxidant capacity, reduced pro-inflammatory cytokine levels and increased anti-inflammatory cytokine level	Chen et al. (2019)
	R3	Alleviate inflammatory cell infiltration, inhibited Th17 and promoted Treg function	Huang et al. (2021)
	N1115	Decreased severity of intestinal tissue injury, cell apoptosis, and proinflammatory cytokines expression	Xun et al. (2022)
*Lactobacillus johnsonii*	UMNLJ22	Suppressed the infiltration of immune cells and secretion of inflammatory cytokines, abrogating ER stress–related cell apoptosis	Zhang et al. (2021)
*Lactobacillus kefiranofaciens*	JKSP109	Enhanced the gut barrier, decreased the level of proinflammatory cytokines and oncocyte proliferation indicators, and increased the expression of terminal deoxynucleotidyl transferase dUTP nick-end labeling-positive tumor epithelial cells	Wang et al. (2022)
*Lactobacillus curvatus*	BYB3	Protected the structural integrity of the intestinal epithelial layer and mucin-secreting goblet cells	Dong et al. (2022)

### 3.1. Probiotics regulate the gut microbiota and increase its diversity

Probiotics can directly increase the relative abundance of *Firmicutes* while decreasing the relative abundance of *Bacteroides* (Cao et al., 2018) to maintain the population of the dominant flora. Furthermore, probiotics can improve adhesion via surface adhesins and surface-expressed fibrinogen, as well as express microbial-associated molecular patterns that interact with pathogenic bacteria for the intestinal epithelial receptor and limit pathogenic bacterial colonization of the gut (Arena et al., 2017). Meanwhile, probiotic supplements hinder the growth of harmful bacteria by consuming metabolic substrates and competing for nutrients. Additionally, probiotics increase the abundance of beneficial bacteria by secreting bacteriocins (Ortiz-Rivera et al., 2017), which can increase the concentration of acetic, lactic, and propionic acids and lower intestinal pH, increase the permeability of the outer membrane of gram-negative bacteria, and directly or indirectly inhibit the growth of pathogenic bacteria (Stavropoulou and Bezirtzoglou, 2020; Ma et al., 2022). Therefore, supplementation with probiotics can effectively maintain homeostasis of the gut microbiota by increasing the abundance of the dominant bacteria.

### 3.2. Probiotics enhance intestinal barrier function

It was proposed that probiotics improve intestinal barrier function via several mechanisms, including increased mucus secretion and upregulation of TJ proteins such as claudin-1, occludin, and ZO-1 via upregulation of MUC2, MUC3, and MUC1 expression in colonic epithelial cells (Kaur et al., 2021). Probiotic-secreted adhesive molecules, such as lipopolysaccharide, polysaccharide A, and peptidoglycan, can stimulate intestinal epithelial cell thickening and increase MUC2 expression. Butyric acid promotes epithelial cell differentiation and mucin synthesis and secretion (Wrzosek et al., 2013). Additionally, *L. reuteri* can promote germinal center-like B cell differentiation to increase the induction and production of PD-1-T follicular helper cell-dependent IgA (Liu et al., 2021). IgA-coated bacteria improved the mucosal barrier better due to their capacity for easy interaction with the host intestine, such as *L. jensenii* and *L. reutri* (Sun et al., 2019; Qi et al., 2021). Furthermore, *L. reutri can* adhere to the surface of Caco-2 or HT29 cells to activate the NF-κB signaling cascade, subsequently upregulating IgA expression and neutralizing bacterial toxins, reducing microbial immunogenicity, and binding to pathogenic microbial surface antigens to prevent the invasion of pathogenic bacteria. *B.bifidum* BB1 improves intestinal epithelial barrier function by connecting to TLR-2 receptors and activating the p38 kinase pathway, which increases TJ expression (Al-Sadi et al., 2021a,b). Similarly, *L. plantarum* NCU116 increases TJ expression by promoting signaling sensors and activators of transcription 3 (STAT3) binding to the promoters of occludin and ZO-1 via EPS (Zhou et al., 2018). *L. fermentum* L930BB and *B. animalis* IM386 are involved in actin cell regulation via the activation of protein kinase C and GTPases as well as phosphatidylinositol-4,5-bisphosphate 3-kinase (PI3K) via TLR-2 receptor/Akt upregulation of the anti-apoptotic pathway. The actin backbone reorganization and the reduction of apoptosis both contribute to the reconstruction of intestinal epithelial cells (Paveljšek et al., 2018). Furthermore, *B. bifidum* increases Caco-2 cell monolayer epithelial resistance, decreases glucose endocytosis, and diminishes LPS-induced colonic damage (Cui et al., 2022). Moreover, *L. plantarum* metabolizes linoleic acid to produce 10-hydroxycis-12-octadecenoic acid, which modulates the GPR40-MEK–ERK pathway and suppresses occludin, ZO-1, and claudin-1 downregulation-induced enhanced intestinal permeability (Miyamoto et al., 2015). *L. plantarum* 12 can prevent colonic damage in mice by increasing colonic TJ protein levels through the suppression of the proliferation of cell nuclear antigen PCNA and enhancement of pro-apoptotic Bax. Furthermore, two soluble proteins (p40 and p75) from L GG were suggested to improve intestinal epithelial homeostasis by preventing cytokine-induced epithelial cell death (Yan et al., 2007). Recent research has found that the probiotic metabolite indolepropionic acid can improve intestinal barrier integrity by interacting directly with the pregnane X receptor 4 (Collins and Patterson, 2020). Therefore, upregulation of TJ expression by probiotics via multiple signaling pathways is the primary mechanism for improving intestinal barrier function.

### 3.3. Probiotics regulate the intestinal immune response

Probiotics can modify immune cells and immunological factors. Meanwhile, probiotics, as innate immune organisms or bacteriophage components, bind to pattern recognition receptors (PRR) on the intestinal mucosa to activate immune cells. *L. plantarum* CBT LP3 exerts anti-inflammatory effects by increasing the number of Tregs, DCs, and Th2 cells while also stimulating the release of anti-inflammatory cytokines such as IL-10 and TGF-β (Kim et al., 2020). *B. adolescentis* EPS activates macrophages via the TLR2-ERK/p38 MAPK signaling cascade, and the macrophage-regulated Treg/Th17 axis suppresses the immunological response (Yu et al., 2019). EPS, proteins, and lipophosphates from *L. acidophilus* LA1 cell walls inhibit NF-κB activation in human HT-29 cells and prevent the release of downstream pro-inflammatory factors (Chen et al., 2013). Immunosuppressive mechanisms are required for intestinal homeostasis, which can be achieved by increasing IL-18 secretion by IECs and the number of Tregs and IL-10-producing T cells. For example, the surface-associated EPS (exopolysaccharide) of *B. longum* 35,624 stimulates the differentiation of naive CD4+ cells into Tregs, which govern effector T cells and exert immunosuppressive effects (Schiavi et al., 2016). LPS-induced NF-κB activation in BV-2 cells can be inhibited by *L. reuteri* NK33 and *B. adolescentis* NK98. Furthermore, probiotic metabolites, particularly SCFAs, can modulate intestinal immune factors, with butyrate activating G protein-coupled receptor 109A signaling to promote Treg differentiation (Couto et al., 2020) and inhibit TLR4/NF-κB signaling pathway-induced pro-inflammatory gene expression (Ghadimi et al., 2020; Zheng et al., 2022). Tryptophan metabolism produces indole compounds that bind to the aromatic hydrocarbon receptor (AHR) and inhibit the production of IFN-γ, IL-6, IL-12, TNF-α, IL-7, and Th17 (Blacher et al., 2017; Chen et al., 2021; Liu et al., 2022), while *L. plantarum* KLDS 1.0386, a strain with high tryptophan metabolizing activity, produces indole-3-acetic acid that further upregulates AHR mRNA expression (Gu et al., 2021; Yang et al., 2021), activating the IL-22/STAT3 signaling pathway and inhibiting inflammatory cytokines production (Shi et al., 2020). Both *Lactobacillus* and *Bifidobacterium* strains can drastically lower adherent-invasive *Escherichia coli* LF82 viability inside macrophages and dendritic cells, decreasing the release of polarizing cytokines linked to the IL-23/Th17 axis (Leccese et al., 2020). NLRP3 components are highly expressed in mice and patients with colitis, and the probiotic MSP inhibits NLRP3 to reduce inflammatory factor production by inhibiting NLRP3-mediated caspase-1 activation (Liu and Wang, 2021; Alkushi et al., 2022). *L. acidophilus* also reduces colonic inflammation through this pathway (Li et al., 2022). In a homeostatic state, metabolites and commensal flora can be recognized by a variety of PRRs, including NOD-like receptors and TLRs, and systematic reviews have shown that the interaction of these receptors is involved in the treatment of UC (Impellizzeri et al., 2015; Kogut et al., 2020). *L. plantarum* inhibits IL-6 and IL-1 secretion to reduce inflammation and improve immunological control by downregulating the expression of JAK, TIRAP, IRAK4, NEMO, and RIP genes in the NF-κB pathway (Aghamohammad et al., 2022; Qian et al., 2022). The LGG effector protein HM0539 of *L. rhamnosus* regulates distal NF-κB activation by lowering TLR4 activation and blocking MyD88 transactivation (Li et al., 2020). *B. infantis* increases T cell-to-Treg conversion by amplifying programmed cell death 1 (PD-1), PD ligand (PD-L1), and the nuclear transcription factor FOXP3, although the mechanism by which PD-1 and PD-L1 are amplified is unknown. Furthermore, it has been proposed that *L. acidophilus* suppresses colitis by modulating immune cells as well as IL-10, thus selectively interfering with endoplasmic reticulum stress. However, it is unclear how probiotics and IL-10 affect endoplasmic reticulum stress, and more research is needed to determine the exact mechanisms of *L. acidophilus.*

## 4. Discussion

The number of studies on the gut microbiota has increased during the last two decades, and scientists are gradually revealing the hidden interactions between the gut microbiota and host. Because the pathophysiology of UC is intrinsically linked to dysbiosis of the gut microbiota, employing probiotics as a therapeutic intervention for UC is justified. Animal trials have demonstrated that probiotics can induce UC remission. The two most dominant genera in the digestive tract, *Bifidobacterium* and *Lactobacillus*, have proven their efficacy in the treatment of UC and are the key constituents in probiotic products currently on the market. Probiotic administration in mice with colitis achieved positive results in terms of symptom relief, tissue improvement, reduction of cellular inflammatory markers, and enhanced expression of tight junction proteins.

Probiotics can be utilized as carriers for genetic engineering and can be altered through genetic engineering. This change can improve the stability of probiotic effects, which may enhance their therapeutic benefits. For example, EcN has been genetically modified to increase the expression of catalase, superoxide dismutase, and endothelial inhibitors (Bi et al., 2022; Zhou et al., 2022), improving its antioxidant effects. The combination of genetic engineering and probiotics for the treatment of UC is widely employed and has a promising future in terms of boosting probiotic efficacy. It is also important to consider the substance of probiotics. Some researchers have employed unique materials as coatings for oral probiotics to preserve their biological activity in the gastrointestinal tract and considerably improve their therapeutic impact (Mauras et al., 2018; Dosoky et al., 2020; Zhou et al., 2022). Fecal transplantation technology is gaining attention in the field of gut microbiota, with two randomized control trials, a meta-analysis, and a review reporting that fecal transplantation (Imdad et al., 2018; Costello et al., 2019; Ooijevaar et al., 2019; Haifer et al., 2022) significantly relieved symptoms of UC, demonstrating the importance of improving the gut microbiota in the treatment of UC. However, fecal transplantation technology is demanding and not routinely performed in clinical practice. Thus, oral probiotic products are still primarily used to maintain the balance of the gut microbiota. Animal experiments have provided substantial evidence regarding the effectiveness of probiotics. However, probiotics take time to colonize the intestine and exert their biological effects (Gao et al., 2019). Hence, hematological therapy and surgery are still preferred for severe and acute UC with serious complications (Kaur et al., 2020). The clinical use of medicines in conjunction with probiotics results in better induction of remission (Tan et al., 2022). The European Society for Parenteral and Enteral Nutrition published the Guidelines for Clinical Nutrition Management of Inflammatory Bowel Disease, which confirm the remission-inducing effect of specific probiotic strains in patients with mild to moderate UC, with the use of *Lactobacillus reuteri* and VSL#3 being recommended (Forbes et al., 2019). In adults with UC, *Bifidobacterium bifidum* powder/capsules, *Lactobacillus* and *Bifidobacterium* triplex tablets, and *Bacillus subtilis* dibacterium enteric-coated capsules are recommended in China. Although there have been no reports of major adverse effects from probiotics, they should be administered with caution in patients with an injured gastrointestinal tract. In clinical studies on patients with UC, the probiotics used, dose and length of treatment regimens, inclusion criteria, and study objectives varied, resulting in convincing evidence regarding the benefits of probiotics. As the pathophysiology of UC and tolerances to probiotics in animals and humans vary, more clinical trials are required to determine the optimal use of probiotics.

## 5. Conclusion

Probiotics are symbiotic organisms that colonize the human gut. The gut microbiota has been widely studied as a critical factor in the occurrence and progression of UC, and probiotics have demonstrated their efficacy in the treatment of experimental UC in mice primarily by correcting gut microbiota dysbiosis, enhancing intestinal mucosa and barrier function, regulating intestinal immune function, and reducing intestinal inflammation. Animal research, however, cannot substitute for clinical trials, and a significant number of high-quality randomized controlled trials are required to establish the efficacy and safety of probiotics. Simultaneously, probiotics have few and mild adverse effects and have a favorable biosafety index. As such, probiotics may be used as an adjuvant treatment of UC. Therefore, clinical trials based on the results of animal experiments are worth exploring, which is the goal of our future research. However, high-quality studies on the efficacy of probiotics in UC with children are extremely limited: there are only two randomized controlled trials and one pilot study in UC. Thus, while it is undoubtedly possible to infer conclusions from evidence acquired in adults, as briefly outlined below, caution must be exercised. Probiotics are now used mostly as adjuvant therapy for UC, and if used as primary therapy, it is necessary to evaluate if the GI environment reduces and inactivates them. Considering the diverse areas of colonization of particular strains and the fact that the primary lesions of UC occur in the colorectum, it is worth investigating if specific strain enemas may be increased to accomplish the effect of boosting effectiveness. For future clinical studies, in addition to clarifying the mechanism of action and dose and duration of treatment, the extent to which probiotics mitigate the adverse effects of existing treatment regimens and the synergistic effects with existing treatment regimens should be explored. For pediatric patients, the low compliance rate and the high rate of missed visits are the main barriers to conducting clinical studies. Moreover, as children are in the growth and development stage, they should be very cautious about the medication of the disease, and the treatment of UC in children with probiotics alone is difficult to achieve.

## Author contributions

QL: conceptualization and funding acquisition. CH, WH, and XW: methodology. CH, WH, XW, and RZ: formal analysis. CH and WH: writing—original draft preparation. CH and WH have contributed equally to this work and share first authorship. RZ and QL: writing—review and editing. XW, RZ, and QL: supervision. All authors have read and agreed to the published version of the manuscript.

## Funding

This research was funded by the Wuxi Medical Innovation Team (Grant No. CXTD2021011).

## Conflict of interest

The authors declare that the research was conducted in the absence of any commercial or financial relationships that could be construed as a potential conflict of interest.

## Publisher’s note

All claims expressed in this article are solely those of the authors and do not necessarily represent those of their affiliated organizations, or those of the publisher, the editors and the reviewers. Any product that may be evaluated in this article, or claim that may be made by its manufacturer, is not guaranteed or endorsed by the publisher.
